# Structured-light control of axion electrodynamics in topological insulator scattering

**DOI:** 10.1038/s41598-026-55269-3

**Published:** 2026-05-28

**Authors:** Muhammad Arfan, Ali Althobaiti, Saad Althobaiti, Igor Meglinski

**Affiliations:** 1 Department of Physics, School of Sciences, School Education Department, Government of Punjab, Faisalabad, 38000 Pakistan; 2 Department of Physics, University of Agriculture, Faisalabad, 38000 Pakistan; 3 Department of Mathematics, College of Science, Taif University, P.O. Box 11099, 21944 Taif, Saudi Arabia; 4 Department of Sciences and Technology, Ranyah University College, Taif University, P.O. Box 11099, 21944 Taif, Saudi Arabia; 5 College of Engineering and Physical Sciences, Aston Institute of Photonic Technologies, Aston University, Birmingham, B4 7ET UK

**Keywords:** Polarisation, Shaped light beams, Electromagnetic scattering, Topological insulators, Lommel beams, Optics and photonics, Physics

## Abstract

Topological insulators exhibit an axion-mediated magnetoelectric response that generates cross-polarised scattering channels strictly forbidden in conventional dielectrics, an optical fingerprint of the topological surface states themselves. Exploiting this fingerprint experimentally requires structured illumination capable of selectively amplifying the cross-polarised channel while suppressing background Mie scattering. Here we show that non-diffracting Lommel beams fulfil this role in a way that symmetric Bessel beams fundamentally cannot. We present the first theoretical treatment of polarised Lommel beam scattering by a topological insulator sphere, extending generalised Lorenz-Mie theory to incorporate the full topological magnetoelectric boundary conditions and deriving closed-form cross-polarised scattering coefficients as a function of the axion angle θ0. The central result is that the Lommel asymmetry parameter *c* provides continuous, tunable control over multipole excitation: by varying *c*, one preferentially drives the multipole orders that couple most strongly to the axion term, amplifying the cross-polarised signal while suppressing the co-polarized background, a capability absent in any cylindrically symmetric beam. At moderate axion coupling (θ0 = π), the cross-polarised-to-co-polarized intensity ratio reaches order 10⁻², well within the detection range of standard polarimetric instrumentation. The inversion protocol for recovering the axion angle shows that the phenomenon is no longer a computational study of a beam-material combination; instead, it is a non-contact optical metrology protocol for axion angle retrieval. The ratio of cross-polarised to co-polarised scattering intensity increases nonlinearly and monotonically with θ0, confirming an unambiguous optical signature of the topological magnetoelectric effect. Circularly polarised Lommel beams further reveal pronounced handedness-dependent scattering asymmetry arising from spin-orbit coupling at the surface states. These results establish a quantitative framework connecting structured light parameters, topological charge, asymmetry, cone angle, and polarisation, to axion electrodynamics, and identify spatial-light-modulator-generated Lommel illumination as a practical, non-contact route to optical characterisation of topological surface states in Bi₂Se₃ and Bi₂Te₃ nanoparticles.

## Introduction

In recent years, the rapid advancement of laser technology has enabled the generation of diverse non-diffracting beams, which maintain their shape over extended distances and recover from obstacles without spreading due to diffraction. These properties, particularly self-reconstruction, are invaluable for applications such as optical trapping and imaging through turbid or random scattering media^1–3^. Among these, the Bessel beam, first proposed by Durnin, stands out as a fundamental type, though an ideal version requires infinite energy and is therefore unrealizable^1^. Nevertheless, quasi-Bessel beams can be practically generated using axicons, making them a powerful tool for advanced microscopy and imaging in complex environments^4–6^.

The interaction of polarised structured beam fields with complex media has attracted considerable attention^7–9^. These kinds of media often tune phase and intensity toward structured beams due to the existence of the topological magnetoelectric (TME) effect^10^. Topological insulators (TIs) are considered among one of these media. TI materials also hold the characteristics of cross-polarisation. The TIs scatter light, but the unique axion-mediated interaction between shaped beams and the surface states of TIs has made these materials a key area of modern optical research. TIs have attracted consideration from both theoretical and experimental sectors due to their unique metallic surface states resulting from topological characteristics^11^. This work describes the axion-based electrodynamics for TIs, stemming the θ-term in the electromagnetic Lagrangian and organising the constitutive relations that support the theoretical modelling^12^. The basic review of TIs and their material realizations is comprehensively discussed in the canonical review^13^.

TI materials preserve the surfaces with insulating and conducting characteristics^14^. The topological surface states in TIs facilitate efficient energy transmission and surface conductivity. The improved constitutive relations for TI are expressed analytically in the context of electric displacement $\:\overrightarrow{D}$ and magnetic field strength $\:\overrightarrow{H}$ as follows: $\:\overrightarrow{D}=\epsilon \overrightarrow{E}+\frac{\alpha\:{\theta\:}_{0}}{\pi\:}\overrightarrow{B}$ and $\:\overrightarrow{H}=\frac{1}{\mu\:}\overrightarrow{B}-\frac{\alpha\:{\theta\:}_{0}}{\pi\:}\overrightarrow{E}$ ^15^. The terms $\:\epsilon$ and $\:\mu\:$ indicate the free-space permittivity and permeability, respectively, while $\:{\theta\:}_{0}$ and $\:\alpha\:$ represent the axion parameter and/or TME polarizability and fine structure constant, respectively^16^. The system remains invariant for permitted values at $\:{\theta\:}_{0}=0$ and/or $\:{\theta\:}_{0}=\pi\:$. Nevertheless, when the time reversal symmetry breaks, then the axion parameter assumes odd integral values of $\:\pi\:$, i.e., $\:{\theta\:}_{0}=\left(2n+1\right)\pi\:$^17^. In TIs, the axion angle $\:{\theta\:}_{0}$ describes a TME coupling that links electric and magnetic fields through an additional **E**·**B** term in the electromagnetic response, leading to polarisation-mixing and helicity-dependent scattering absent in conventional dielectrics^18^.

The most common physical phenomenon in nature is the scattering of light. A structured light beam yields unparalleled control over light’s fundamental characteristics, including phase profile, polarisation states, and intensity distribution. Typically, structured light is defined as light beams with significant intriguing amplitude and polarisation distributions^19^. The extensive variety of potential applications for light scattering is enabled by tailoring multiple degrees of freedom of structured light^20^. It includes particle manipulation, remote sensing, and quantum information technologies. One potentially constructive type of structured light is non-diffracting light beams that propagate independently without changing their intensity distribution in the transverse direction. The concept of orbital angular momentum (OAM)^21^ possessed by light beams, first introduced by Allen et al., is basic to beam shaping, a property we explore to analyse the scattering response of the TI sphere.

A Bessel beam offers a clean and well-understood probe of scattering systems: its cylindrical symmetry, fixed angular spectrum, and single topological charge make it analytically tractable and practically reproducible^22^. Within the generalized Lorenz-Mie theory (GLMT) framework, the beam shape coefficients of a Bessel beam are determined entirely by the topological charge v and the half-cone angle α, once these two parameters are fixed, the angular spectrum is fully determined and no further continuous degree of freedom remains^23^. Yet this same constraint is a fundamental limitation when probing the topological magnetoelectric effect in TI spheres. Because the cross-polarised scattering coefficients induced by the axion coupling are mode-dependent, different multipole orders coupling to the TME term with different strengths, selectively amplifying the axion signature over the co-polarised Mie background requires a beam whose angular spectrum can be shaped continuously to preferentially drive the most axion-sensitive multipoles. Recent work on structured-light interactions with resonant particles has established that engineering the incident beam profile provides exactly this kind of selective multipole control^24,25^: by redistributing energy among multipole orders through beam shape coefficients, one can enhance or suppress specific scattering channels in ways inaccessible to symmetric illumination. A standard Bessel beam cannot exploit this principle, its angular spectrum is fixed, whereas non-diffracting Lommel beams break this constraint directly. Defined as a linear superposition of Bessel modes weighted by the complex asymmetry parameter *c*^26^, Lommel beams reduce exactly to a vortex Bessel beam when *c* = 0, but for *c* ≠ 0 they develop a tunable, azimuthally asymmetric angular spectrum that couples energy selectively into chosen multipole orders^27–29^. The contrast with Bessel illumination is not merely a matter of degree: as demonstrated in recent vector Lommel scattering studies, varying c continuously redistributes the energy among multipole orders in a way that no parameter of a Bessel, Gaussian, or Laguerre-Gaussian beam can replicate^29^. This continuous degree of freedom makes the Lommel beam a uniquely suited probe for the mode-selective physics of the topological magnetoelectric effect, and, more broadly, positions structured asymmetric beams as a new tool for resolving topology-sensitive scattering responses in quantum materials^30^.

Various analytical frameworks have been proposed to characterize the scattering characteristics of TI geometries. Electromagnetic radiation illuminated by a time-reversal symmetric TI circular cylinder and symmetric broken TI sphere has been examined^31,32^. Findings suggest that the scattering width may serve to validate the fine structure constant. A systematic study of the scattering response of the TI sphere is conducted^33^. A metamaterial perfect electromagnetic conducting (PEMC) cylinder coated with TI material was investigated using a Gaussian beam source^34^. Plane wave incidence on a TI cylinder coated with various metamaterials has been explored^35^. It is shown that radial and azimuthal modes of vortex beams can be used to tune the scattering response of a TI cylinder coated with different metamaterials^36^.

The asymmetric angular spectrum can be constructed by employing the other structured beam families, including superpositions of Laguerre–Gaussian (LG) beam modes with dissimilar topological charges. The LG beam modes can be generated utilizing Hermite-Gaussian (HG) modes and vice versa. LG beam mode superpositions would involve fine-tuned modal weights lacking clearer physical insight. So, we chose Lommel beams for more than just their asymmetry characteristics; there are other practical and physical reasons as well.

First, Lommel beams offer a closed-form, analytically controllable description of an asymmetric angular spectrum resulting from a tractable superposition of Bessel beam modes. This enables a direct connection between the configuration and degree of asymmetry and the selective stimulation of cross-polarised fields, a relationship that is more tangible in arbitrary beam superpositions such as LG mode combinations, where the angular spectrum does not have a simple analytical representation. Second, Lommel beams retain significant features of quasi-diffraction-free propagation and a well-defined transverse wavevector distribution, which is lacking in generic asymmetric vortex beams. This makes sure that the electromagnetic interaction with the spherical scatterer is controlled by a predictable and stable angular spectrum over the distance of propagation. This is essential for segregating the mode-selective coupling phenomena. Third, we want to make it clear that we are not using Lommel beams to infer uniqueness from its nature. Instead, we employed it right here to generate a fundamental and physically transparent model that visibly shows the working of the angular spectrum asymmetry system. Lastly, we want to point out that ideal Lommel beams embrace infinite energy, just like that of ideal Bessel beams. However, quasi-diffraction-free Lommel beams with finite-energy approximations compared to ideal Lommel beams are developed well and employed throughout this work, removing any practical implications.

To date, there has been no theoretical study of polarised Lommel beams scattering by TI materials, making this study very comprehensive to report this gap. The significance of scattering Lommel beams stems from both analytical and numerical considerations. In most of the scattering situations, the cylinder is lit up immediately, which makes it easier to work with. However, when the object is not directly illuminated and the incident field interacts with the interface first, the challenges are more complex. The light sources directly irradiate the objects, whether cylindrical or spherical, according to these scattering schemes. So, these scenarios make it easier to work with. In contrast, when the surfaces are not directly illuminated and the incident light field interacts with the interfaces, the studies become much more complex.

In this work, our approach employs the framework of GLMT, applying it to explore the unmapped non-diffracting category of structured beams toward the renowned class of metamaterial structures. This study provides a comprehensive analysis of the scattering characteristics of the TI scatterer. We present both analytical and simulated results. As Lommel beams with many of the configuration parameters introduced may influence the scattering characteristics in the context of both scattering intensity and scattering efficiency in ways not reachable with other structured beams. More beam control parameters yield additional particle information, which pertains to particle manipulation and measurement technologies. Configuration parameters for Lommel beams enable control over beam intensity and spatial propagation, making them a valuable tool for analyzing theoretical insights related to electromagnetic field interactions in topological photonics, quantum optics, tunable shaped light engineering, and optoelectronic technologies.

The methodological extension of GLMT to structured non-diffracting Lommel beams (NDLBs) is a straightforward way. The theoretical insight lies in the beam’s asymmetry parameter, which provides a knob to increase or decrease specific multipole moments. This reveals a substantial result: the TME effect does not just align the scattering characteristics but infuses helicity-based asymmetries that cannot be delivered with symmetric-shaped beams. We explore that tuning parameter c can repress the Mie scattering response from the bulk scattering while resonantly amplifying the cross-polarised TME signature-efficiently.

## Methods

**Fig. 1:**
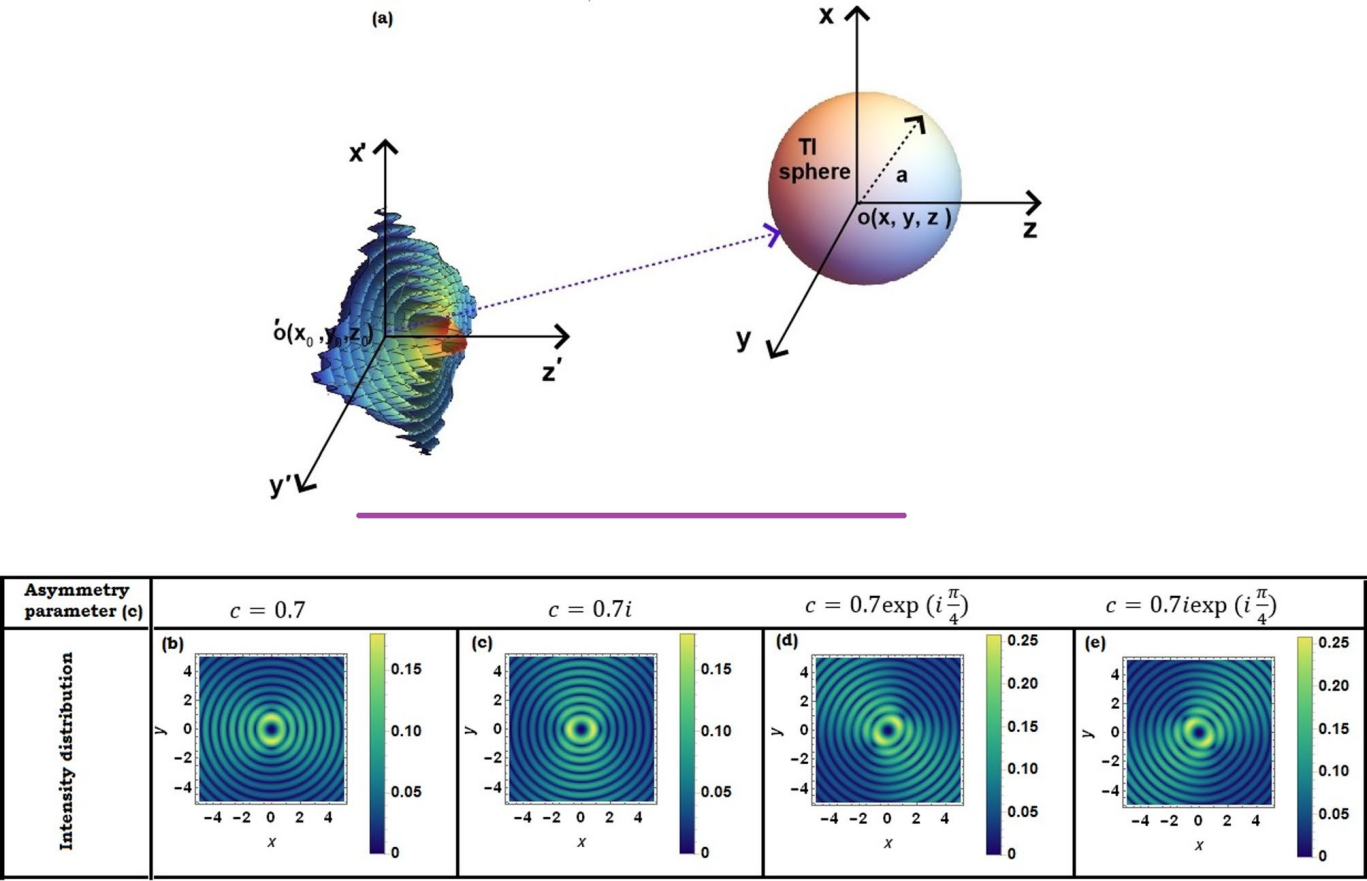
(**a**) Schematic of a topological insulator sphere of radius *a* illuminated by a non-diffracting Lommel beam propagating along the *z*-direction; (**b–e**) transverse intensity distributions of the Lommel beam for various asymmetry parameters: (**b**) *c* = 0.7, (**c**) *c* = 0.7*i*, (**d**) *c* = 0.7exp(*iπ*/4), (**e**) *c* = 0.7*i*·exp(*iπ*/4).

Figure 1(a) displays the interaction model of the problem. Right here, in free space, the NDLB field illuminates the TI sphere. The field propagation is along the *\:z* direction, whereas its polarisation is about the *\:x* direction. The surrounding medium around the TI sphere is homogeneous, non-conducting, non-magnetic, and lossless. For TI, a represents the radius of the sphere. The coordinates of the TI sphere are *\:O(x,y,z)*. The $\:{O}^{{\prime\:}}({x}^{{\prime\:}},{y}^{{\prime\:}},{z}^{{\prime\:}})$ represents the axis coordinates of the NDLB, while $\:{O}^{{\prime\:}}\left({x}_{0}{,\:y}_{0}{,z}_{0}\right)$ designates its center coordinates. For easiness, the $\:{e}^{i\omega\:t}$ term, which characterizes time-dependence, is omitted all over the analytical calculations.

Figure 1(b-e) displays the intensity profiles of the Lommel beam for various asymmetry parameters. When $\:c\ne\:0$, the beam intensity manifests a ring of an oval shape. However, for complex values of the asymmetry factor, the ring rotates with a shifting of the phase angle of $\:{90}^{0}$. By including a factor of $\:\left(\frac{\pi\:}{4}\right)$ in the value of c, the density plots for Lommel beams take the form of oval-shaped rings arranged diagonally. Increasing the value of *\:c* results in changing the beam’s intensity.

Nondiffracting beams are a solution of the well-known Helmholtz equation $\:{\nabla\:}^{2}E+\:{\left(\frac{\omega\:}{c}\right)}^{2}E=0$. Here, Maxwell’s equations are solved to form the renowned Helmholtz equation. In cylindrical coordinate systems, the required non-diffracting Lommel beam can be achieved as a superposition of the Bessel beam modes^26^, i.e.,

1 $$\:\psi\:\left(r,\varphi\:,z\right)=\sum\:_{p=0}^{\infty\:}{\left(-1\right)}^{p}{c}^{2p}{J}_{v+2p}\left({k}_{t}r\right){e}^{i(v+2p)\varphi\:}{(e}^{i{k}_{z}z})$$

where $\:{J}_{v+2p}(.)$ is the Bessel function of the first kind where *\:v+2p* represents its order. *\:c* indicates asymmetry factor. The topological charge of the Lommel beam is denoted by the term v. $\:\varphi\:$ expresses the phase angle with $\:\varphi\:={arctan}\left(\frac{y}{x}\right)$. The wave number is expressed as $\:k=\sqrt{{k}_{t}^{2}+{k}_{z}^{2}}=\frac{2\pi\:}{\lambda\:}$, where $\:{k}_{t}=ksin\alpha\:$ and $\:{k}_{z}=kcos\alpha\:$ are transverse and longitudinal wave vectors. $\:\alpha\:$ is known as the beam half-cone angle.

The amplitude distribution of the NDLB field can be characterized using (1) as

2 $$\:\:\psi\:\left(r,\varphi\:,z\right)=\left\{\begin{array}{c}\begin{array}{c}c=0=v\:\:\:\:\:\:\:\:\:\:\:\:\:\:\:\left(Bessel\:beams\right)\\\:c=0\ne\:v\:\:\:\:\:\:\:\:\:\:\:\:\:\:\left(Lommel\:beams\right)\\\:c\ne\:0=v\:\:\:\:\:\:\:\:\:\:\:\:\:\:\left(Lommel\:beams\right)\end{array}\\\:c\ne\:0\ne\:v\:\:\:\:\:\:\:\:\:\:\:\:\:\:\left(Lommel\:beams\right)\end{array}\right.\:$$

In GLMT^37^, the beam fields of an arbitrarily structured beam can be expressed analytically in the context of vector spherical wave functions (VSWFs) and beam shape coefficients (BSCs).

3 $$\:{\boldsymbol{E}}^{inc}=\sum\:_{n=1}^{\infty\:}\sum\:_{m=-n}^{n}{c}_{n}^{pw}\left[{{g}_{n,TM}^{m}\mathbf{N}}_{mn}^{\left(1\right)}+i{{g}_{n,TE}^{m}\mathbf{M}}_{mn}^{\left(1\right)}\right]$$

4 $$\:{\boldsymbol{H}}^{inc}=-\left(i\frac{k}{\omega\:}\right)\sum\:_{n=1}^{\infty\:}\sum\:_{m=-n}^{n}{c}_{n}^{pw}\left[{g}_{n,TM}^{m}{\mathbf{M}}_{mn}^{\left(1\right)}+i{g}_{n,TE}^{m}{\mathbf{N}}_{mn}^{\left(1\right)}\right]$$

with $$\:{c}_{n}^{pw}={(-i)}^{n+1}\frac{2n+1}{n(n+1)}$$.

where the $\:{\mathbf{M}}_{mn}^{\left(1\right)}$ and $\:{\mathbf{N}}_{mn}^{\left(1\right)}$ characterize the VSWFs of the first kind. Furthermore, $\:{g}_{n,TM}^{m}$ and $\:{g}_{n,TE}^{m}$ designate the expansion coefficients and/or BSCs. TE stands for transverse electric, while TM represents transverse magnetic. These BSCs possess information regarding the beam illumination and various polarisations states of the incident non-diffracting Lommel beam. The expansion coefficients for different polarisations can be given^23,29^

5 $$\begin{aligned} &\:{g}_{n,TM\text{}}^{m,\text{}x-LP}=\left(\frac{1}{2}\right){i}^{m+1}{\left(-1\right)}^{\frac{m-\left|m\right|}{2}}\:\frac{\left(n-m\right)!}{\left(n+\left|m\right|\right)!}\\& *{\sum\:}_{p=0}^{\infty\:}{\left(-1\right)}^{p}{c}^{2p}\left\{\begin{array}{c}{J}_{m-\nu\:-2p-1}\left({k}_{t}{\rho\:}_{0}\right){e}^{-i\left(m-\nu\:-2p-1\right){\varphi\:}_{0}}\left[m{\pi\:}_{n}^{m}\left(cos\alpha\:\right)+\frac{{\tau\:}_{n}^{m}\left(cos\alpha\:\right)}{cos\alpha\:}\right]\\\:+{J}_{m-\nu\:-2p+1}\left({k}_{t}{\rho\:}_{0}\right){e}^{-i\left(m-\nu\:-2p+1\right){\varphi\:}_{0}}\left[m{\pi\:}_{n}^{m}\left(cos\alpha\:\right)-\frac{{\tau\:}_{n}^{m}\left(cos\alpha\:\right)}{cos\alpha\:}\right]\end{array}\right\}{e}^{i{k}_{z}{z}_{0}} \end{aligned}$$

6 $$\begin{aligned} &\:{g}_{n,TE\text{}}^{m,\text{}x-LP}=\left(\frac{-i}{2}\right){i}^{m+1}{\left(-1\right)}^{\frac{m-\left|m\right|}{2}}\:\frac{\left(n-m\right)!}{\left(n+\left|m\right|\right)!}\\& *{\sum\:}_{p=0}^{\infty\:}{\left(-1\right)}^{p}{c}^{2p}\left\{\begin{array}{c}{J}_{m-\nu\:-2p-1}\left({k}_{t}{\rho\:}_{0}\right){e}^{-i\left(m-\nu\:-2p-1\right){\varphi\:}_{0}}\left[m\frac{{\pi\:}_{n}^{m}\left(cos\alpha\:\right)}{cos\alpha\:}+{\tau\:}_{n}^{m}\left(cos\alpha\:\right)\right]\\\:-{J}_{m-\nu\:-2p+1}\left({k}_{t}{\rho\:}_{0}\right){e}^{-i\left(m-\nu\:-2p+1\right){\varphi\:}_{0}}\left[m\frac{{\pi\:}_{n}^{m}\left(cos\alpha\:\right)}{cos\alpha\:}-{\tau\:}_{n}^{m}\left(cos\alpha\:\right)\right]\end{array}\right\}{e}^{i{k}_{z}{z}_{0}}\end{aligned}$$

7 $$\begin{aligned} &\:{g}_{n,TM\text{}}^{m,\text{}y-LP}=\left(\frac{i}{2}\right){i}^{m+1}{\left(-1\right)}^{\frac{m-\left|m\right|}{2}}\:\frac{\left(n-m\right)!}{\left(n+\left|m\right|\right)!}\\& *{\sum\:}_{p=0}^{\infty\:}{\left(-1\right)}^{p}{c}^{2p}\left\{\begin{array}{c}{J}_{m-\nu\:-2p-1}\left({k}_{t}{\rho\:}_{0}\right){e}^{-i\left(m-\nu\:-2p-1\right){\varphi\:}_{0}}\left[m{\pi\:}_{n}^{m}\left(cos\alpha\:\right)+\frac{{\tau\:}_{n}^{m}\left(cos\alpha\:\right)}{cos\alpha\:}\right]\\\:-{J}_{m-\nu\:-2p+1}\left({k}_{t}{\rho\:}_{0}\right){e}^{-i\left(m-\nu\:-2p+1\right){\varphi\:}_{0}}\left[m{\pi\:}_{n}^{m}\left(cos\alpha\:\right)-\frac{{\tau\:}_{n}^{m}\left(cos\alpha\:\right)}{cos\alpha\:}\right]\end{array}\right\}{e}^{i{k}_{z}{z}_{0}}\end{aligned}$$

8 $$\begin{aligned} &\:{g}_{n,TE\text{}}^{m,\text{}y-LP}=\left(\frac{1}{2}\right){i}^{m+1}{\left(-1\right)}^{\frac{m-\left|m\right|}{2}}\:\frac{\left(n-m\right)!}{\left(n+\left|m\right|\right)!}\\& *{\sum\:}_{p=0}^{\infty\:}{\left(-1\right)}^{p}{c}^{2p}\left\{\begin{array}{c}{J}_{m-\nu\:-2p-1}\left({k}_{t}{\rho\:}_{0}\right){e}^{-i\left(m-\nu\:-2p-1\right){\varphi\:}_{0}}\left[m\frac{{\pi\:}_{n}^{m}\left(cos\alpha\:\right)}{cos\alpha\:}+{\tau\:}_{n}^{m}\left(cos\alpha\:\right)\right]\\\:+{J}_{m-\nu\:-2p+1}\left({k}_{t}{\rho\:}_{0}\right){e}^{-i\left(m-\nu\:-2p+1\right){\varphi\:}_{0}}\left[m\frac{{\pi\:}_{n}^{m}\left(cos\alpha\:\right)}{cos\alpha\:}-{\tau\:}_{n}^{m}\left(cos\alpha\:\right)\right]\end{array}\right\}{e}^{i{k}_{z}{z}_{0}}\end{aligned}$$

9 $$\begin{aligned} &\:{g}_{n,TM\text{}}^{m,\:\:\:R-CP}={i}^{m+1}{\left(-1\right)}^{\frac{m-\left|m\right|}{2}}\:\frac{\left(n-m\right)!}{\left(n+\left|m\right|\right)!}\\& {\sum\:}_{p=0}^{\infty\:}{\left(-1\right)}^{p}{c}^{2p}{J}_{m-\nu\:-2p-1}\left({k}_{t}{\rho\:}_{0}\right){e}^{-i\left(m-\nu\:-2p-1\right){\varphi\:}_{0}}\left[m{\pi\:}_{n}^{m}\left(cos\alpha\:\right)+\frac{{\tau\:}_{n}^{m}\left(cos\alpha\:\right)}{cos\alpha\:}\right]{e}^{i{k}_{z}{z}_{0}}\end{aligned}$$

10 $$\begin{aligned} &\:{g}_{n,TE\text{}}^{m,\:\:\:R-CP}={i}^{m}{\left(-1\right)}^{\frac{m-\left|m\right|}{2}}\:\frac{\left(n-m\right)!}{\left(n+\left|m\right|\right)!}\\& {\sum\:}_{p=0}^{\infty\:}{\left(-1\right)}^{p}{c}^{2p}{J}_{m-\nu\:-2p-1}\left({k}_{t}{\rho\:}_{0}\right){e}^{-i\left(m-\nu\:-2p-1\right){\varphi\:}_{0}}\left[{\tau\:}_{n}^{m}\left(cos\alpha\:\right)+m\frac{{\pi\:}_{n}^{m}\left(cos\alpha\:\right)}{cos\alpha\:}\right]{e}^{i{k}_{z}{z}_{0}}\end{aligned}$$

11 $$\begin{aligned} &\:{g}_{n,TM\text{}}^{m,\:\:\:L-CP}={i}^{m+1}{\left(-1\right)}^{\frac{m-\left|m\right|}{2}}\:\frac{\left(n-m\right)!}{\left(n+\left|m\right|\right)!}\\& {\sum\:}_{p=0}^{\infty\:}{\left(-1\right)}^{p}{c}^{2p}{J}_{m-\nu\:-2p+1}\left({k}_{t}{\rho\:}_{0}\right){e}^{-i\left(m-\nu\:-2p+1\right){\varphi\:}_{0}}\left[m{\pi\:}_{n}^{m}\left(cos\alpha\:\right)-\frac{{\tau\:}_{n}^{m}\left(cos\alpha\:\right)}{cos\alpha\:}\right]{e}^{i{k}_{z}{z}_{0}}\end{aligned}$$

12 $$\begin{aligned} &\:{g}_{n,TE\text{}}^{m,\:\:\:L-CP}={i}^{m}{\left(-1\right)}^{\frac{m-\left|m\right|}{2}}\:\frac{\left(n-m\right)!}{\left(n+\left|m\right|\right)!}\\& {\sum\:}_{p=0}^{\infty\:}{\left(-1\right)}^{p}{c}^{2p}{J}_{m-\nu\:-2p+1}\left({k}_{t}{\rho\:}_{0}\right){e}^{-i\left(m-\nu\:-2p+1\right){\varphi\:}_{0}}\left[{\tau\:}_{n}^{m}\left(cos\alpha\:\right)-m\frac{{\pi\:}_{n}^{m}\left(cos\alpha\:\right)}{cos\alpha\:}\right]{e}^{i{k}_{z}{z}_{0}}\end{aligned}$$

where $\:{\pi\:}_{n}^{m}\left(.\right)$ and $\:{\tau\:}_{n}^{m}\left(.\right)$ designate the (associated) Legendre functions of order m and degree *n*.

Similarly, the scattered and internal electromagnetic fields are expressed as

13 $$\:{\boldsymbol{E}}^{sca}=\sum\:_{n=1}^{\infty\:}\sum\:_{m=-n}^{n}{c}_{n}^{pw}\left[{a}_{mn}^{sca}{\mathbf{N}}_{mn}^{\left(3\right)}+i{b}_{mn}^{sca}{\mathbf{M}}_{mn}^{\left(3\right)}\right]$$

14 $$\:{\boldsymbol{H}}^{sca}=-\left(i\frac{k}{\omega\:}\right)\sum\:_{n=1}^{\infty\:}\sum\:_{m=-n}^{n}{c}_{n}^{pw}\left[{a}_{mn}^{sca}{\mathbf{M}}_{mn}^{\left(3\right)}+i{b}_{mn}^{sca}{\mathbf{N}}_{mn}^{\left(3\right)}\right]$$

15 $$\:{\boldsymbol{E}}^{int}=\sum\:_{n=1}^{\infty\:}\sum\:_{m=-n}^{n}{c}_{n}^{pw}\left[{c}_{mn}^{sca}{\mathbf{N}}_{mn}^{\left(1\right)}+i{d}_{mn}^{sca}{\mathbf{M}}_{mn}^{\left(1\right)}\right]$$

16 $$\:{\boldsymbol{H}}^{int}=-\left(i\frac{{m}_{r}k}{\omega\:}\right)\sum\:_{n=1}^{\infty\:}\sum\:_{m=-n}^{n}{c}_{n}^{pw}\left[{c}_{mn}^{sca}{\mathbf{M}}_{mn}^{\left(1\right)}+i{d}_{mn}^{sca}{\mathbf{N}}_{mn}^{\left(1\right)}\right]$$

The superscript 3 is used for scattered fields, and the spherical Hankel function of the first kind $\:{h}_{n}^{1}\left(kr\right)$ is involved. Whereas, the spherical Bessel function $\:{j}_{n}\left({k}_{1}r\right)$ is included for transmitted electromagnetic fields.

The unknown scattering coefficients, i.e., $\:{a}_{mn}^{sca}$ and $\:{b}_{mn}^{sca}$ of scattered electromagnetic fields, can be found by applying the following boundary conditions (BCs) as

17 $$\:\widehat{\boldsymbol{r}}\times\:\left({\boldsymbol{E}}^{inc}+{\boldsymbol{E}}^{sca}-{\boldsymbol{E}}^{int}\right)=0$$

18 $$\:\widehat{\boldsymbol{r}}\times\:\left({\boldsymbol{H}}^{inc}+{\boldsymbol{H}}^{sca}-{\boldsymbol{H}}^{int}\right)=0$$

The substitution of electromagnetic fields for the above-mentioned BCs yields the relationship of the expansion coefficients of incident, scattered, and internal fields as

19 $$\:{a}_{mn}^{sca}={g}_{n,TM}^{m}{a}_{n}^{TI}+{g}_{n,TE}^{m}{b}_{n}^{TI}$$

20 $$\:{b}_{mn}^{sca}={g}_{n,TM}^{m}{c}_{n}^{TI}+{g}_{n,TE}^{m}{d}_{n}^{TI}$$

Considering the TI sphere, the coefficients $\:{a}_{n}^{TI}$ and $\:{b}_{n}^{TI}$ represent the cross-polarised scattering field coefficients, while $\:{c}_{n}^{TI}$ and $\:{d}_{n}^{TI}$ designate the cross-polarised internal field coefficients.

The cross-polarised scattering field coefficients can be connected to the internal field coefficients as

$$\:{a}_{n}^{TI}=\frac{{\left[{m}_{r}x{j}_{n}\left({m}_{r}x\right)\right]}^{{\prime\:}}}{{m}_{r}{\left[x{h}_{n}^{\left(1\right)}\left(x\right)\right]}^{{\prime\:}}}{d}_{n}^{TI};\:{b}_{n}^{TI}=\frac{{j}_{n}\left({m}_{r}x\right)}{{h}_{n}^{\left(1\right)}\left(x\right)}{c}_{n}^{TI}.$$

The coefficients $\:{c}_{n}^{TI}$ and $\:{d}_{n}^{TI}$ can be expressed in the context of internal coefficients $\:{c}_{n}$ and $\:{d}_{n}$ as

$$\:{c}_{n}^{TI}=\left(\stackrel{\sim}{\alpha\:}{\chi\:}_{2}\right){d}_{n};\:{d}_{n}^{TI}=\left(\stackrel{\sim}{\alpha\:}{\chi\:}_{1}\right){c}_{n}.$$

where $\:{c}_{n}$ and $\:{d}_{n}$ designate the classical scattering coefficients within the Lorenz-Mie theory.

21 $$\:{c}_{n}=\frac{{j}_{n}\left(x\right){\mu\:}_{1}{\left[x{h}_{n}^{\left(1\right)}\left(x\right)\right]}^{{\prime\:}}-{\mu\:}_{1}{\left[x{j}_{n}\left(x\right)\right]}^{{\prime\:}}{h}_{n}^{\left(1\right)}\left(x\right)}{{j}_{n}\left({m}_{r}x\right){\mu\:}_{1}{\left[x{h}_{n}^{\left(1\right)}\left(x\right)\right]}^{{\prime\:}}-{\mu\:}_{b}{\left[{m}_{r}x{j}_{n}\left({m}_{r}x\right)\right]}^{{\prime\:}}{h}_{n}^{\left(1\right)}\left(x\right){\beta\:}_{1}}$$

22 $$\:{d}_{n}=\frac{{\mu\:}_{1}{j}_{n}\left(x\right){\left[x{h}_{n}^{\left(1\right)}\left(x\right)\right]}^{{\prime\:}}{m}_{r}-{\mu\:}_{1}{h}_{n}^{\left(1\right)}\left(x\right){\left[x{j}_{n}\left(x\right)\right]}^{{\prime\:}}{m}_{r}}{{\mu\:}_{b}{j}_{n}\left({m}_{r}x\right){\left[x{h}_{n}^{\left(1\right)}\left(x\right)\right]}^{{\prime\:}}{m}_{r}^{2}{\beta\:}_{2}-{\mu\:}_{1}{h}_{n}^{\left(1\right)}\left(x\right){\left[{m}_{r}x{j}_{n}\left({m}_{r}x\right)\right]}^{{\prime\:}}}$$

where, $\:x\:=\:ka$ symbolizes the size parameter. $\:{m}_{r}$ indicates the relative refractive index. $\:{\mu\:}_{b}$ and $\:{\mu\:}_{1}$ denote permeabilities regarding free space and the TI sphere, respectively. Furthermore, $\:{\beta\:}_{1}$ and $\:{\beta\:}_{2}$ stand for auxiliary functions that are interlinked with the axion angle $\:{\theta\:}_{0}$ of the TI sphere as $\:{\beta\:}_{1}=1+{\stackrel{\sim}{\alpha\:}}^{2}{\chi\:}_{1}$ and $\:{\beta\:}_{2}=1+{\stackrel{\sim}{\alpha\:}}^{2}{\chi\:}_{2}$ while $\:\stackrel{\sim}{\alpha\:}=\left(\frac{\alpha\:{\theta\:}_{0}}{\pi\:}\right)\sqrt{\frac{{\mu\:}_{1}}{{\epsilon\:}_{1}}}\:$, and, $\:{\chi\:}_{1}$ and $\:{\chi\:}_{2}$ can be written as

23 $$\:{\chi\:}_{1}=\frac{{\mu\:}_{b}{m}_{r}^{2}{j}_{n}\left({m}_{r}x\right){\left[x{h}_{n}^{\left(1\right)}\left(x\right)\right]}^{{\prime\:}}}{{\left[x{h}_{n}^{\left(1\right)}\left(x\right)\right]}^{{\prime\:}}{\mu\:}_{b}{m}_{r}^{2}{j}_{n}\left({m}_{r}x\right)-{\mu\:}_{1}{h}_{n}^{\left(1\right)}\left(x\right){\left[{m}_{r}x{j}_{n}\left({m}_{r}x\right)\right]}^{{\prime\:}}}$$

24 $$\:{\chi\:}_{2}=\frac{{\mu\:}_{b}{h}_{n}^{\left(1\right)}\left(x\right){\left[{m}_{r}x{j}_{n}\left({m}_{r}x\right)\right]}^{{\prime\:}}}{{\left[x{h}_{n}^{\left(1\right)}\left(x\right)\right]}^{{\prime\:}}{\mu\:}_{1}{j}_{n}\left({m}_{r}x\right)-{\mu\:}_{b}{h}_{n}^{\left(1\right)}\left(x\right){\left[{m}_{r}x{j}_{n}\left({m}_{r}x\right)\right]}^{{\prime\:}}}$$

Far-field scattering intensity (FSI) is the angular distribution of the scattered power in the far-zone (far-field). FSI varies with the scattering angle. The far-field scattering intensity (FSI) and scattering efficiency using the scattering field coefficients are respectively represented as

25 $$\:FSI(\theta\:,\varphi\:)={\left|{S}_{1}\left(\theta\:,\varphi\:\right)\right|}^{2}+{\left|{S}_{2}\left(\theta\:,\varphi\:\right)\right|}^{2}$$

26 $$\:{Q}_{sca}=\left(\frac{2}{{\rho\:}^{2}}\right)\sum\:_{n=1}^{\infty\:}\sum\:_{m=-n}^{n}\frac{2n+1}{n\left(n+1\right)}\left({\left|{a}_{mn}^{sca}\right|}^{2}+{\left|{b}_{mn}^{sca}\right|}^{2}\right)$$

where $\:{S}_{1}$ and $\:{S}_{2}$ characterize scattering amplitudes^38^.

27 $$\:{S}_{1}\left(\theta\:,\varphi\:\right)=\sum\:_{n=1}^{\infty\:}\sum\:_{m=-n}^{n}\frac{2n+1}{n(n+1)}[m{a}_{mn}^{sca}{\pi\:}_{n}^{\left|m\right|}\left(cos\theta\:\right)+i{b}_{mn}^{sca}{\tau\:}_{n}^{\left|m\right|}\left(cos\theta\:\right)]{e}^{im\varphi\:}$$

28 $$\:{S}_{2}\left(\theta\:,\varphi\:\right)=\sum\:_{n=1}^{\infty\:}\sum\:_{m=-n}^{n}\frac{2n+1}{n(n+1)}[{a}_{mn}^{sca}{\tau\:}_{n}^{\left|m\right|}\left(cos\theta\:\right)+im{b}_{mn}^{sca}{\pi\:}_{n}^{\left|m\right|}\left(cos\theta\:\right)]{e}^{im\varphi\:}$$

## Results

The problem under study, i.e., polarisation-based scattering characteristics of NDLBs, is studied. In this part, the NDLB field is employed to investigate its interaction with the topological insulator sphere. This section includes the simulation of the required factors for NDLB-based scattered intensity and polarisation degree of the scattered beam. The impacts of the parameters for Lommel beam configuration are also analyzed. Here, various parameters such as the beam topological charge, asymmetry parameters, polarisation states, axion angle, half-cone angle, and beam center locations are numerically analyzed and then discussed. Lommel beam with operating wavelength of $\:\lambda\:\:=\:632.8\:nm$ is employed. The other parameters are set as beam topological charge *\:v=1*, asymmetry parameter *\:c=0.2*, beam half-cone angle $\:\alpha\:={2}^{}$, and axion angle of topological insulator $\:{\theta\:}_{0}=4\pi\:$ otherwise declared in the simulation results.

**Fig. 2:**
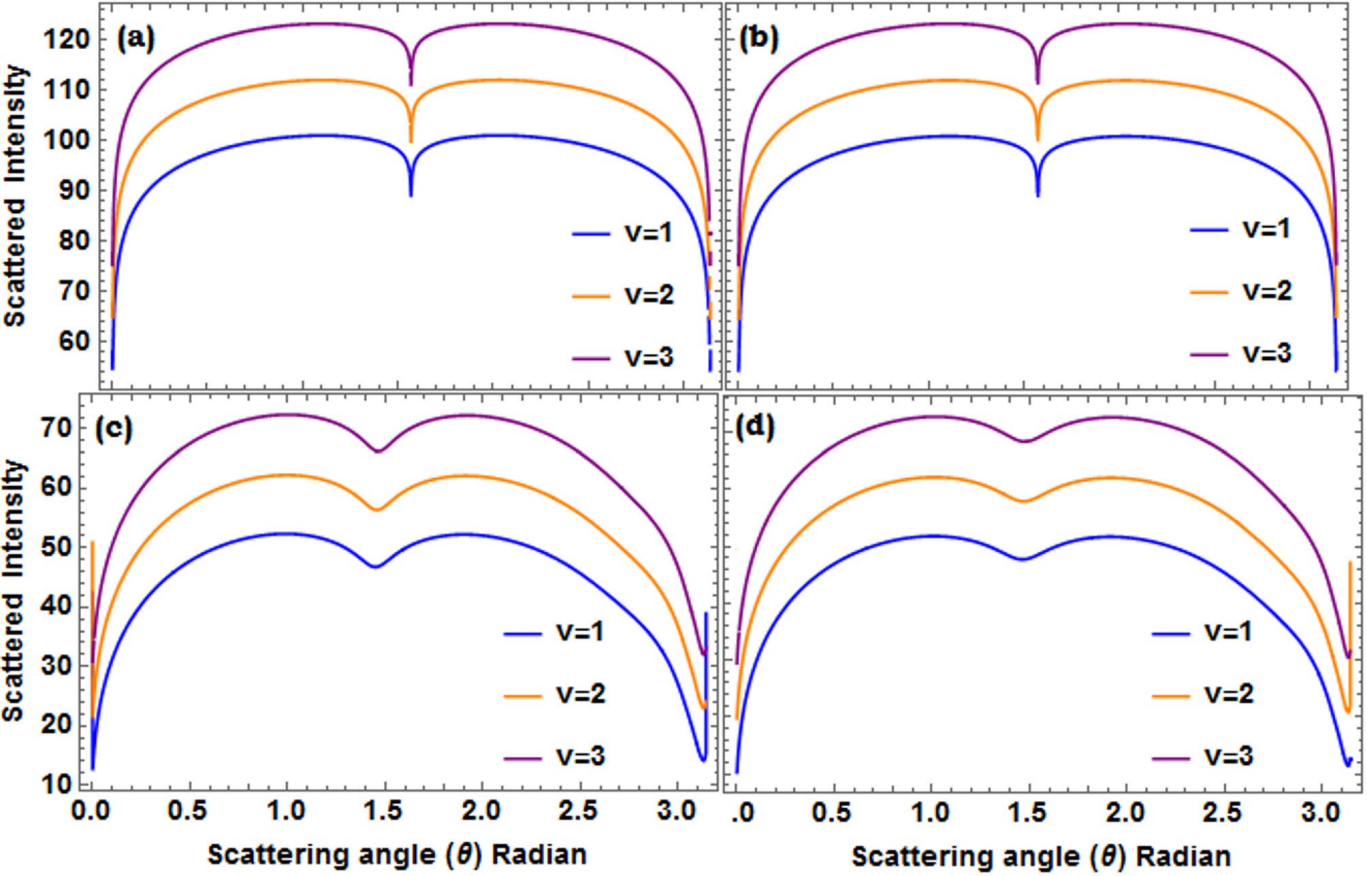
FSI with varying topological charge v = 1,2, and 3 for different polarisation states. Panels (a–d) correspond to x-linear, y-linear, Left circularly polarised (LCP), and Right circularly polarised (RCP), respectively. The remaining simulation parameters are the asymmetry parameter c = 0.2, axion angle $\:{\theta\:}_{0}$=4π, beam half-cone angle $\:\alpha\:={2}^{}$, and beam center coordinates $\:\left({x}_{0}{,\:y}_{0}{,z}_{0}\right)=(1.0\lambda\:,1.0\lambda\:,1.0\lambda\:)$.

**Fig. 3:**
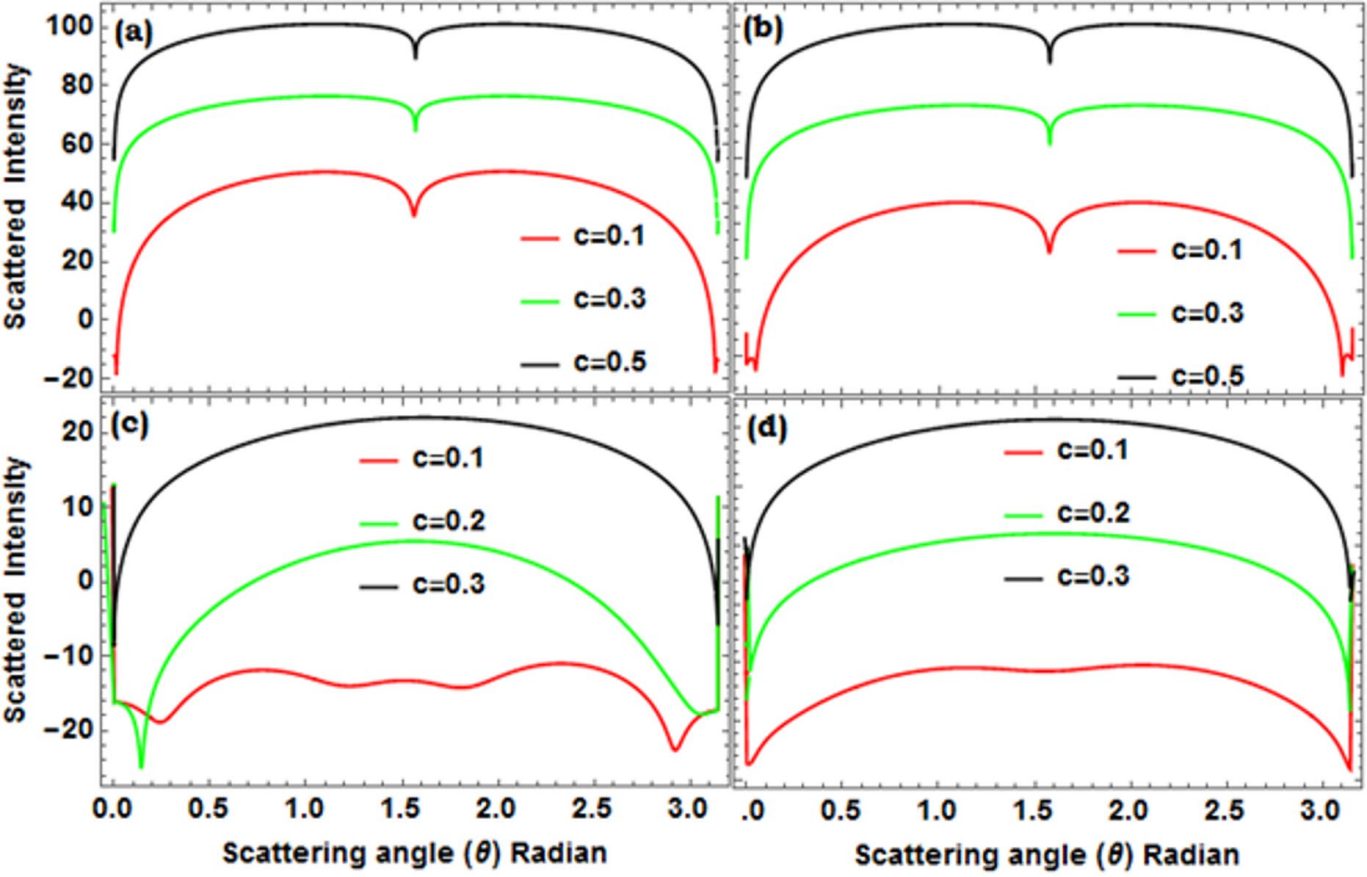
FSI with varying asymmetry parameter c = 0.1,0.3, and 0.5 for different polarisation states. Panels (a–d) correspond to x-linear, y-linear, LCP, and RCP, respectively. The remaining simulation parameters are the beam’s topological charge v = 1, beam’s half-cone angle $\:\alpha\:={2}^{}$, axion angle $\:{\theta\:}_{0}$=4*π*, and beam center coordinates $\:\left({x}_{0}{,\:y}_{0}{,z}_{0}\right)=(1.0\lambda\:,1.0\lambda\:,1.0\lambda\:)$.

**Fig. 4:**
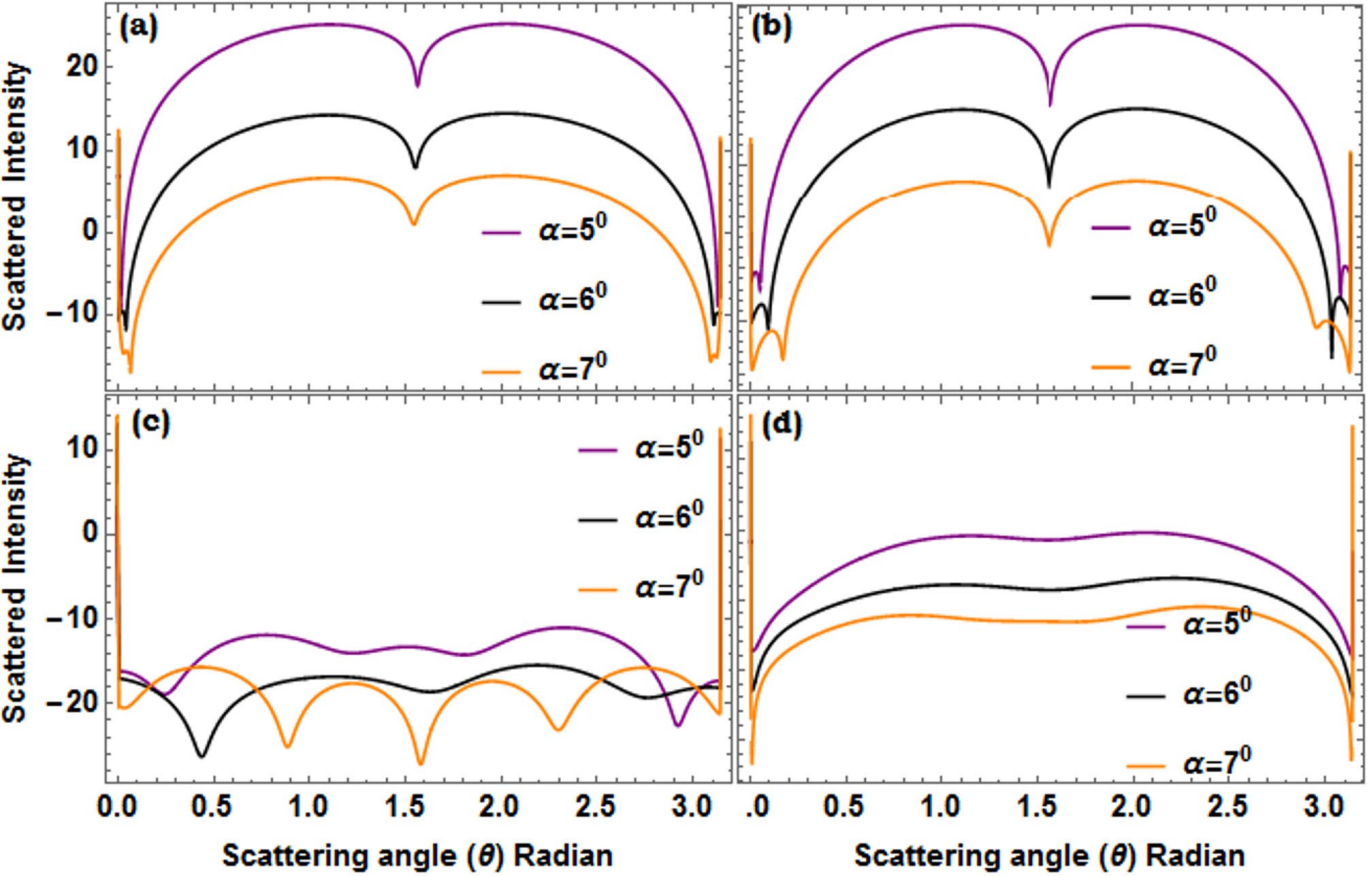
FSI with varying beam’s half-cone angle $\:{\upalpha\:}={5}^{}$,$\:\:{6}^{}$,and $\:{7}^{}$ for different polarisation states. Panels (a–d) correspond to x-linear, y-linear, LCP, and RCP, respectively. The remaining simulation parameters are the beam’s topological charge v = 1, asymmetry parameter c = 0.2, axion angle $\:{\theta\:}_{0}$=4*π*, and beam center coordinates $\:\left({\mathrm{x}}_{0}{,\:\mathrm{y}}_{0}{,\mathrm{z}}_{0}\right)=(1.0{\uplambda\:},1.0{\uplambda\:},1.0{\uplambda\:})$.

Figures 2, 3 and 4 shows the effects of beam parameters, including topological charge, asymmetry parameter, and half-cone angle. Figure 2 displays the influences of the beam’s topological charge, i.e., v = 1,2,3, against various polarisation states regarding the scattered intensity distributions. When a TI sphere scatters structured light (Lommel beam), the topological charge v of the beam affects the intensity of the scattered Lommel beam in a way that also depends on the polarisation states of the light. This is mainly because of the electromagnetic interaction of the various factors. The foremost factor among them is the helical phase interaction, i.e., $\:{e}^{iv\varphi\:}$, it imparts the OAM toward the scattered light intensity. The incident polarisation states, such as x-linear, y-linear, left-circular, and right-circular, mainly tune the beam field of the Lommel beam. The changing polarisation states of the Lommel beam impact the interference phenomena with OAM, so this in turn influences the scattered intensity. NDLBs with lower topological charge generally excite lower-order multipoles. On increasing the topological charge of the Lommel beams, the higher-order multipoles are excited with diverse boosted field distributions. Furthermore, the intensity of the scattered light beam increases as the intensity of the incident beam rises in relation to the topological charge of the NDLB.

Figure 3 shows the impact of the beam’s asymmetry parameter on FSI. The asymmetry parameter c in a NDLB governs the off-axis vortex profile and conical wavefront configuration, thereby modifying the phase distribution and spatial intensity illumination on the TI sphere. A nonzero c changes the azimuthal structure of the incoming light beam field, which changes the phase gradient and intensity profile. These inconsistent illuminations of the Lommel beam are due to $\:c\ne\:0$ exciting multipoles considering the TI sphere. The dependence of the intensity distribution of NDLB is determined by the asymmetry parameter *\:c*. It means for $\:c\ne\:0\:\wedge\:\:\in\:\mathrm{R}$ (real valued), the intensity pattern shows symmetry about the optical *\:x*-axis, while for $\:c\ne\:0\:\wedge\:\:\in\:\mathrm{C}$ (complex valued), the beam intensity flips along the *\:y*-axis. Such a type of response can be seen in Fig. 1(b-e). The topological magnetoelectric effect response to this uneven excitation is by changing the asymmetry factor for different values such as 0.1, 0.2, and 0.3, and this affects the polarisation conversion spherical scatters in different directions. Furthermore, there are structural changes in the beam’s profile, such as tilting and shifting, along with an increase in the asymmetry parameter.

Figure 4 exhibits the influence of half-cone angle on FSI. The half-cone angle (α) of NDLB for different values, like as $\:{5}^{}$,$\:\:{6}^{}$,and $\:{7}^{}$ impacts the degree of non-diffraction and transverse wavevector component, so influencing the balance of parts of momentum. For structured beams, the spot diameter is expressed as $\:\frac{\pi\:}{kt}$, where *\:kt* denotes the transverse part regarding the wave vector phase constant. Its analytical expression is given as $\:kt=ksin\alpha\:$, so, aftermath the diameter transforms as $\:\frac{{\uplambda\:}}{2\mathrm{s}\mathrm{i}\mathrm{n}{\upalpha\:}}$. According to the aforementioned expression, an increase in the beam’s half-cone angle (α) results in an increase towards $\:sin\alpha\:$; subsequently, the diameter of the spot size decreases. Furthermore, increasing the half-cone angle causes the beam waist radius to reduce, which in turn causes the interactions with the spherical scatterer of TI to become robust and more focused, in lieu of the other way around. Far-field scattered intensity tunes by increasing the half-cone angle of NDLB, which exposes a higher portion of the spherical region to the incoming light beam field and consequently illuminates a larger surface area of the TI sphere.

**Fig. 5:**
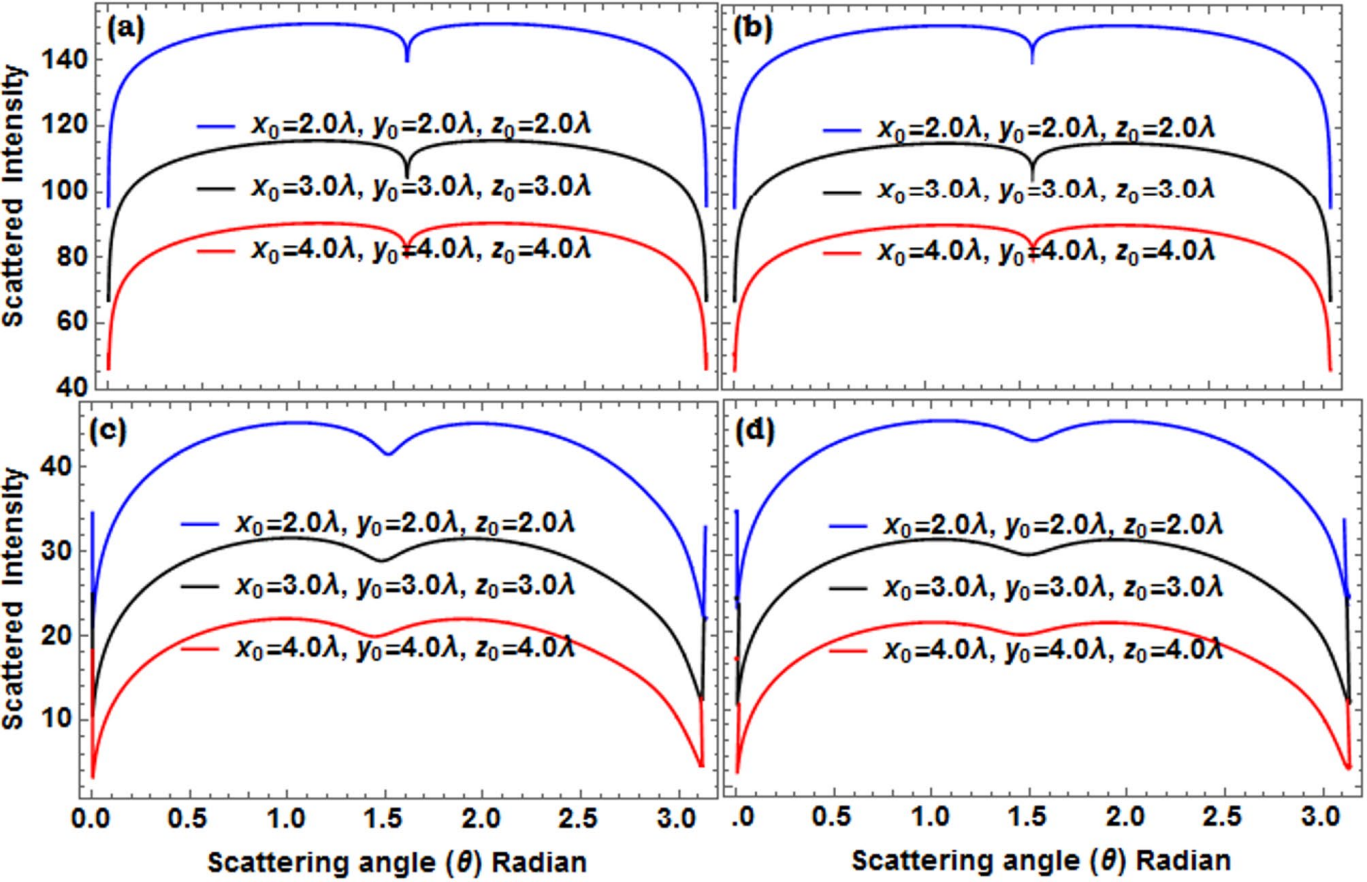
FSI with varying beam’s center coordinates for different polarisation states. Panels (a–d) correspond to x-linear, y-linear, LCP, and RCP, respectively. The remaining simulation parameters are the beam’s topological charge v = 1, asymmetry parameter *c* = 0.2, beam’s half-cone angle $\:\alpha\:={2}^{}$, and axion angle $\:{\theta\:}_{0}$=4*π*.

**Fig. 6:**
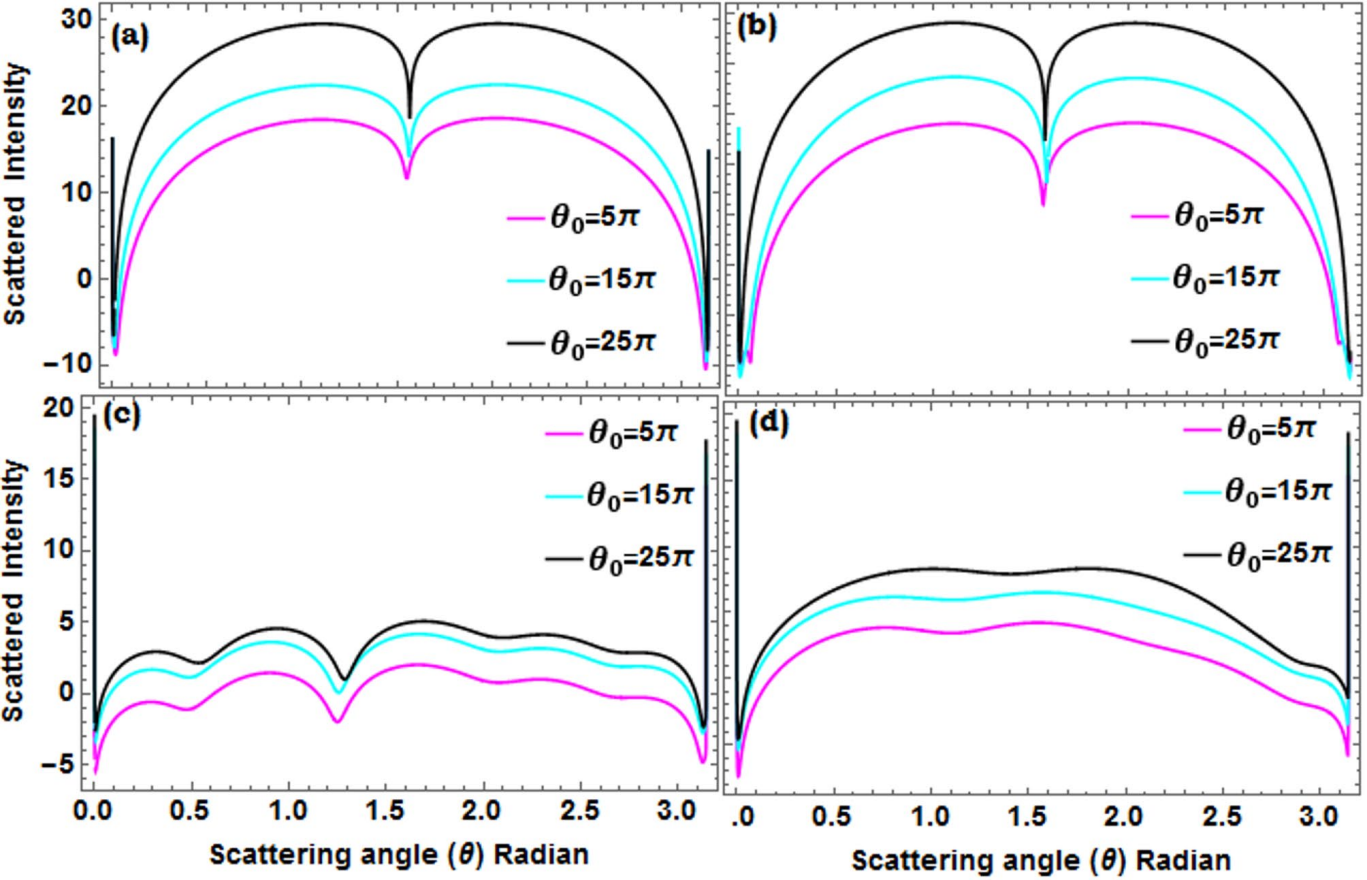
FSI with varying axion angle for different polarisation states. Panels (a–d) correspond to x-linear, y-linear, LCP, and RCP, respectively. The remaining simulation parameters are the beam’s topological charge v = 1, asymmetry parameter c = 0.2, beam’s half-cone angle $\:\alpha\:={2}^{}$, and beam center coordinates $\:\left({x}_{0}{,\:y}_{0}{,z}_{0}\right)=(1.0\lambda\:,1.0\lambda\:,1.0\lambda\:)$.

Figures 5 and 6 displays the influences of TI/geometry effects, such as beam center locations and axion angle. Figure 5 shows the impact of beam center locations on FSI. By deviating from the locations of the beam center, the symmetrical characteristics of beam illumination are disrupted, yielding an irregular distribution of electromagnetic multipoles that are increased selectively subject to the direction of the offset regarding the TI sphere. There are direction-dependent scattering curves that are aligned with the optical axis when dealing with linear polarisations, because the off-centering couples to the orientation of the incident beam field. The scattered field distribution also undergoes polarisation-sensitive variations as a result of the coupling between spin and orbit when circular polarisations are applied, as the transverse displacement interaction with the spin-based phase profile of the NDLB. Asymmetric illumination, caused by shifting the center of the spherical surface of TI’s away from the locations of the beam’s center, reduces scattered intensity in the far-field by interfering destructively with the scattered field pattern.

Figure 6 exhibits the impact of the axion angle $\:\left({\theta\:}_{0}\right)$ on the FSI of Lommel’s beam. It can be seen that the axion angle $\:\left({\theta\:}_{0}\right)$ is a key factor in unfolding the scattering response of the TI scatterer. In the classical frame of GLMT, only the co-polarised scattering coefficients, i.e., $\:{a}_{n}$ and $\:{b}_{n}$, involve towards the scattered types of electromagnetic fields; nevertheless, considering the TI scatterer, two extra coefficients, $\:{a}_{n}^{TI}$ and $\:{b}_{n}^{TI}$, known as cross-polarised fields components, are also involved in the analytical expressions of scattered fields due to the axion parameter of TI. Increasing the axion parameter results in exciting the TME, which interlinks electromagnetic scattered fields. The distinctive polarisation-sensitive response is produced by varying the axion angle $\:{\theta\:}_{0}$ of spherical scatterer TI, which results in obvious intensity differences for various polarisation states, irrespective of the spatial structure of the incident Lommel beam field.

For conventional material structures, the cross-polarised field coefficients are zero, while for the case of TIs, the cross-polarised components are involved due to the TME phenomena. It is also important to note that the TME term is not included in the scattering and internal field coefficients, whereas it plays a significant role for the cross-polarised coefficients. The influence of the TME term is quite obvious for the graphical results.

Considering Figs. 3 and 4, and 6, the negative values do not represent physical and/or measurable scattered intensity. As FSI is defined in Eq. (25) of the manuscript using the GLMT. To better illustrate the wide dynamic range of the scattering response, the plotted quantity corresponds to the logarithmic representation for all figures corresponding to the scattered intensity in the far-field region. Consequently, a combination of both positive and negative values in different figures is a direct consequence of logarithmic scaling and does not represent any physical inconsistency.

The sharp variations in scattered intensity occur around 0 and 3 radians considering Figs. 2, 3, 4, 5 and 6 (c–d). These differences are physical and are not caused by the numerical artifacts. These generate due to interference phenomena and multipolar resonances in GLMT. The scattering field coefficients are very responsive towards the configuration parameters of the TI medium, i.e., the axion angle and the diffraction-free Lommel beam asymmetry parameter, which govern the amplitude factor as well as the phase profile of the excited electromagnetic multipoles. At specific values of configuration parameters of diffraction-free Lommel beams, robust inter-modal field coupling occurs between adjacent multipolar orders in addition to the swift phase transitions, and these lead to boosting and/or attenuation of the intensity distribution, resulting in abrupt scattering characteristics. These types of resonance-based responses are well-known in various shaped beam scatterings.

Besides, the difference between LCP and RCP in Figs. 2, 3, 4, 5 and 6 (c–d) is a genuine polarisation-based phenomenon. This evolves from axion electrodynamics in TIs, expressed by the term “$\:{\theta\:}_{0}$(**E**.**B**),” which modifies the constitutive relations to involve electromagnetic field coupling. So, electromagnetic responses turned out to be intrinsically coupled, yielding handedness-based scattering characteristics that lift the degeneracy between LCP and RCP and result in circular dichroism–like asymmetry features in FSI.

**Fig. 7:**
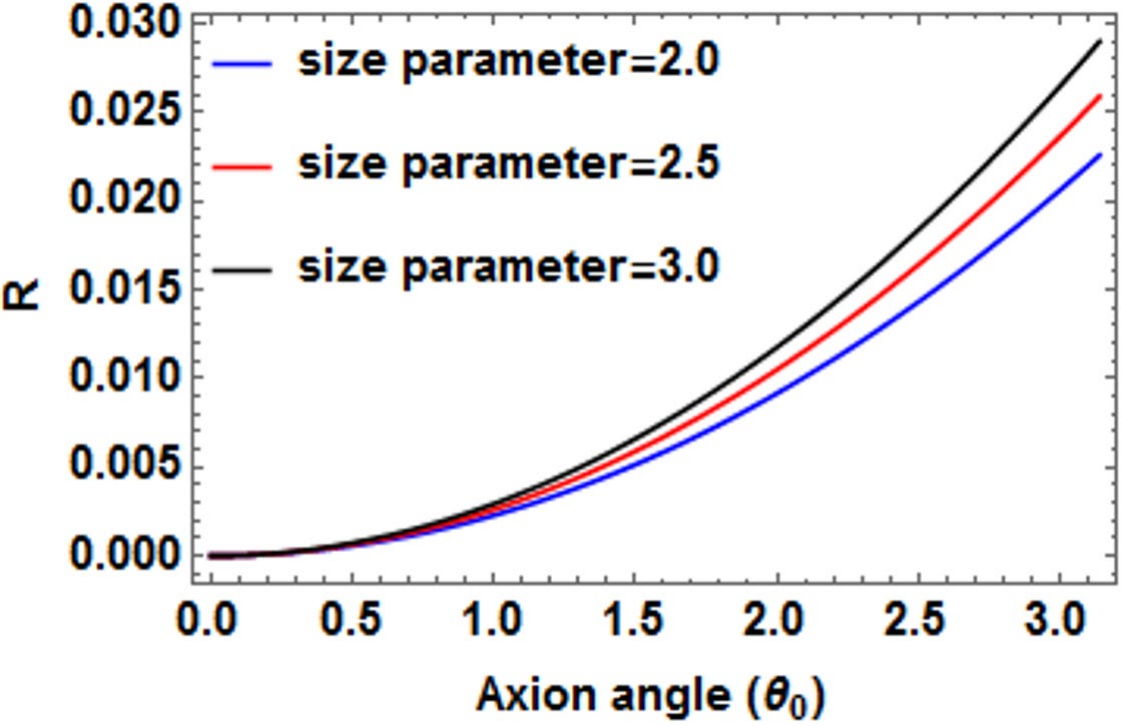
The ratio of cross-polarised to co-polarised scattering intensity $\:\mathrm{R}\left({{\uptheta\:}}_{0},\:\mathrm{c},\:\mathrm{k}\mathrm{a}\right)=\frac{\sum\:_{n=1}^{{N}_{max}}\sum\:_{m=-n}^{n}{\left|{a}_{mn,cross}\right|}^{2}}{\sum\:_{n=1}^{{N}_{max}}\sum\:_{m=-n}^{n}{\left|{a}_{mn,co}\right|}^{2}}$ where the sum runs over all multipoles retained in the GLMT expansion (*n* = 1 to N_max_, with N_max_ chosen for converged scattering coefficients) as a function of axion angle $\:{\theta\:}_{0}$ for different size parameters, i.e., x=2.0, 2.5, and 3.0 in the NDLB scattering by a TI sphere. The ratio increases with axion angle $\:{\theta\:}_{0}$, reaching up to values of the order of 10⁻², representing a detectable cross-polarised contribution induced by the TME effect.

To quantify the practical significance of the TME-induced cross-polarisation, we evaluate the ratio of cross-polarised to co-polarised scattering intensity, $\:\mathrm{R}\left({{\uptheta\:}}_{0},\:\mathrm{c},\:\mathrm{k}\mathrm{a}\right)=\frac{\sum\:_{n=1}^{{N}_{max}}\sum\:_{m=-n}^{n}{\left|{a}_{mn,cross}\right|}^{2}}{\sum\:_{n=1}^{{N}_{max}}\sum\:_{m=-n}^{n}{\left|{a}_{mn,co}\right|}^{2}}$, versus axion angle $\:{\theta\:}_{0}$ for representative size parameters in Fig. 7. This quantity measures the total cross-polarised power scattered by the particle relative to the total co-polarised power, independent of which multipole carries the dominant contribution. At $\:{\theta\:}_{0}$ $\:\approx\:$ *π*, this ratio reaches values of order 10⁻² for the dominant dipole modes (*n* = 1), indicating that the cross-polarised contribution constitutes of a few percent of the total scattered power. This is substantially above the noise floor of modern polarimetric detection systems, which routinely resolve polarisation extinction ratios exceeding 10⁻⁴. The cross-polarised signal therefore represents an experimentally accessible signature of the axion coupling, distinguishable from the co-polarised background without requiring specialised instrumentation. For $\:{\theta\:}_{0}$ → 0, R → 0, recovering the conventional dielectric limit, confirming that the cross-polarised response is uniquely attributable to the TME effect rather than geometric or refractive index contrast.

The cross-polarised-to-co-polarised intensity ratio reaches up to the order of 10⁻², and this range is detectable for modern photodetectors. They can detect and resolve the electromagnetic signals that are ~ 10² to ~ 10^3^ times feebler compared to that of the main co-polarised signal. Various noise sources, like thermal noise (TN), shot noise (SN), and background scattering noise (BSN), influence the capability of signal detection. Nevertheless, within the limited frame of the SN, the signal-to-noise ratio (SNR) varies as SNR$\:\propto\:\sqrt{signal\:intensity}$. It means that stable signals reaching order 10⁻² remain reasonably measurable. Practically, typical experimental methods like polarisation filtering, signal averaging, and locking-in detection can significantly enhance the reasonable detection of this type of feeble cross-polarised signals. In optical scattering experiments, these approaches are often employed to achieve weak signals out of noisy backgrounds, allowing optical community to precisely analyse the characteristics of diverse materials and/or biological samples that display nominal scattering response.

**Fig. 8:**
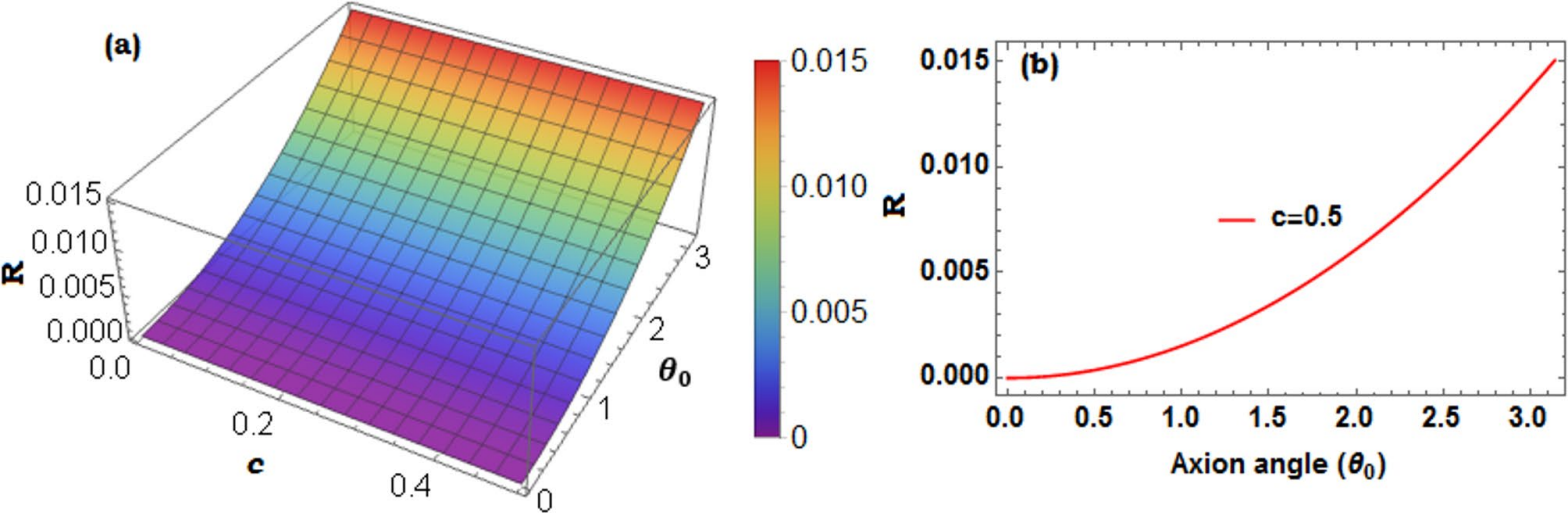
Inversion protocol for recovering the axion angle $\:{\theta\:}_{0}$: (a) Calibration surface regarding the polarimetric ratio as a combined function of the diffraction-free Lommel beam asymmetry c and the axion angle $\:{\theta\:}_{0}$ of TI, and (b) Inversion curve R for a fixed Lommel beam asymmetry parameter c, demonstrating retrieval of the axion angle $\:{\theta\:}_{0}$ from a measured polarimetric ratio R term. A fixed baseline of R(c, θ₀) exists at c = 0, and it generates from symmetry-dependent TME coupling; furthermore, increasing the c parameter improves otherwise weak coupling.

Figure 8a represents the calibration surface plot of the polarimetric ratio R as a joint function of the diffraction-free Lommel beam asymmetry c and the axion angle $\:{\theta\:}_{0}$ of the TI. The ratio R increases monotonically with both the aforementioned parameters, reflecting the establishment of the TME coupling. This monotonic response strengths a one-to-one communication between polarimetric ratio R and axion term $\:{\theta\:}_{0}$ for a Lommel beam of known c parameter, establishing the base of an optical calibration plot for retrieval of axion-angle. For NDLB with known value of c parameter, a measured value of R exclusively achieves the axion angle $\:{\theta\:}_{0}$, enabling non-contact optical recovery of the TME coupling.

Figure 8b shows the inversion curve R($\:{\theta\:}_{0}$) for a fixed beam asymmetry parameter c, demonstrating the experimental recovery protocol. Through this calibration curve, a detected polarimetric ratio R exceptionally evaluates the corresponding axion angle $\:{\theta\:}_{0}$ of the TI. For $\:{\theta\:}_{0}$ → 0, R → 0, achieving the conventional dielectric response and validating that the cross-polarised signal generates from the TME effect. At c = 0 there is a symmetric-beam baseline from intrinsic TME coupling; c modulates which channels contribute and increases R away from c = 0. The monotonic growth of R with θ₀, and the role of the asymmetry parameter c in opening additional conversion channels, are preserved, while the absolute values shift modestly.

**Fig. 9:**
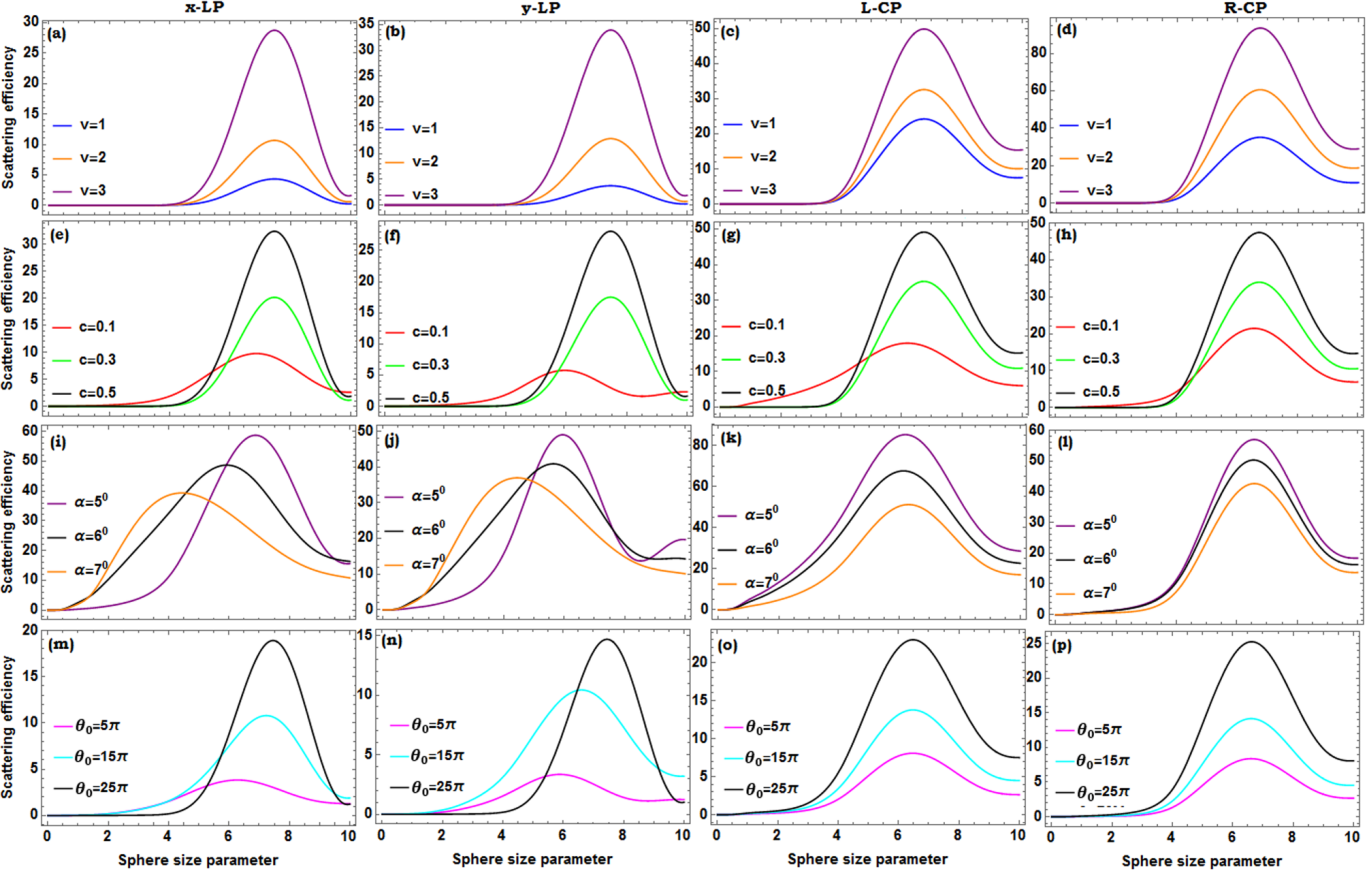
Impact of NDLB configuration parameters and TI axion angle on scattering efficiency of TI sphere for diverse polarisation states. Rows designate (**a-d**) beam’s topological charge, (**e-h**) asymmetry parameter, (**i-l**) half-cone angle, and (**m-p**) axion angle. Left-to-right column entries are for linear-x, linear-y, circular-left, and circular-right polarisation.

Figure 9 shows the scattering efficiency $\:\left({Q}_{sca}\right)$ of a TI sphere illuminated by the Lommel beam across four polarisation states, i.e., x-linear polarisation (x-LP), y-linear polarisation (y-LP), left-circular polarisation (L-CP), and right-circular polarisation (R-CP). Contrary to traditional dielectric spherical scatterers, the cross-polarised scattering field components also contribute toward $\:{Q}_{sca}$. The topological charge of the Lommel beam is set as $\:(v=1,\:2,\:\&3)$. The interplay between the Lommel beam’s topological charge and the TI sphere makes the scattering efficiency particularly sensitive. Various field modes are involved for varying the beam’s topological charge. Increasing *\:v* results in enhanced electromagnetic scattering and holding the non-diffraction characteristics very well. The beam’s half-cone angle is varied, and the graphical results indicate that the scattering efficiency changes due to the nonuniform spatial distribution of the NDLB. Increasing the beam’s axion angle, the beam’s divergence strengthens, yielding a dispersion of the beam’s energy for the dimensionless size parameter.

By applying external fields, time-reversal symmetry is broken, and the TME influences and modifies the system. It is defined in the context of the Lagrangian term as $\:L=\frac{1}{8\pi\:}\left(\epsilon {\boldsymbol{E}}^{2}-\frac{1}{\mu\:}{\boldsymbol{B}}^{2}\right)+\left(\frac{\alpha\:}{4{\pi\:}^{2}}\right){\theta\:}_{0}\left(\boldsymbol{E}.\boldsymbol{B}\right)$, where $\:\boldsymbol{E}$ and $\:\boldsymbol{B}$ characterize the electric field and magnetic flux density, respectively. For a traditional dielectric, $\:{\theta\:}_{0}=0,$ while for $\:{\theta\:}_{0}=\pi\:$, it results in TIs. Changing $\:{\theta\:}_{0}$ for 5$\:\pi\:$,15$\:\pi\:$, and 25$\:\pi\:$ and an applied external electric field induces a magnetization as $\:\mathbf{M}\propto\:{\theta\:}_{0}\:\mathbf{E}$, whereas the TME term and changing magnetic field strength induce a polarisation as $\:\mathbf{P}\propto\:{\theta\:}_{0}\:\mathbf{B}$. NDLB with increased axion angle holds substantially pronounced spatial beam field gradients. So, this part exhibits much boosted interaction with the surface of the TI and the subsequent polarisation and/or magnetization due to the TME.

The asymmetry parameter c alters the beam’s spot size and shaped ring oval-type, and it governs the degree of freedom towards the intensity distribution of Lommel’s beam. Additionally, variations in the beam’s asymmetry parameter can distribute the scattered intensity. Since the c parameter controls the characteristics of the Lommel beam field, varying it influences the light’s spatial profile, which in turn affects scattering efficiency.

**Fig. 10:**
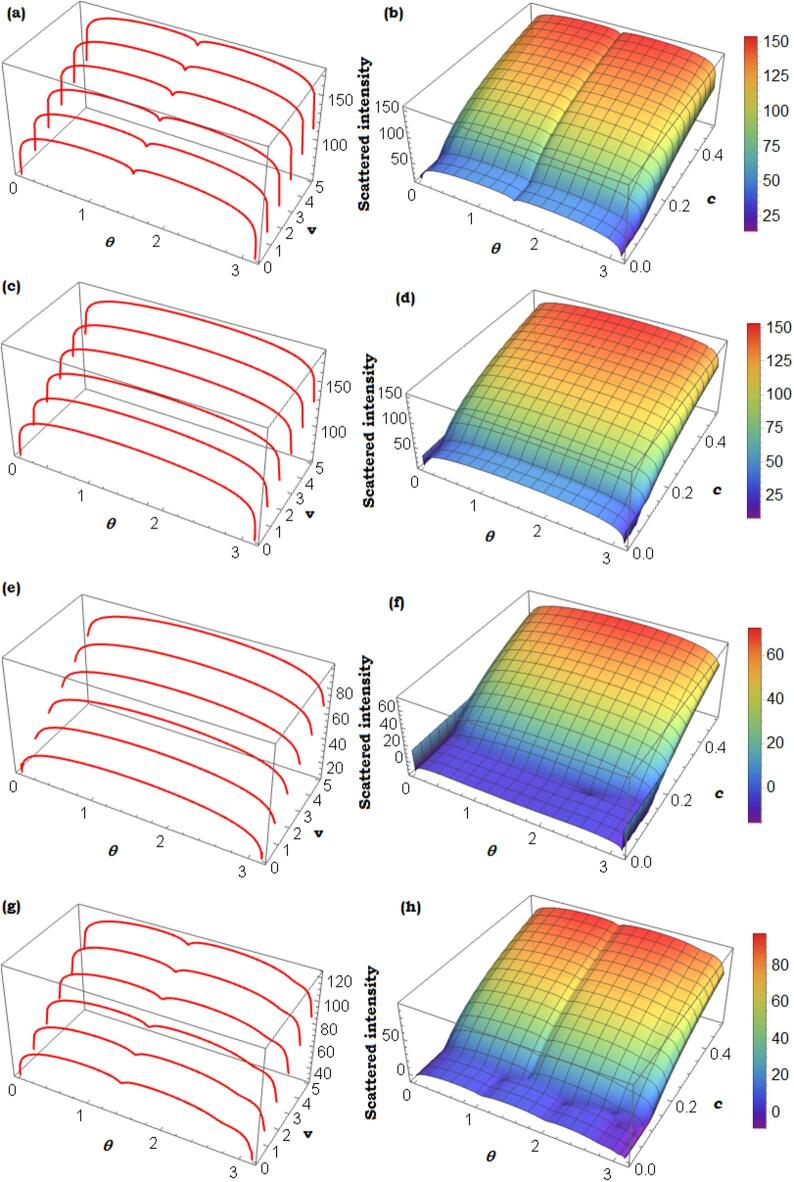
Scattered intensity in the far-field region for TI scatterer under illumination of Lommel beam on varying the scattering angle versus topological charge and asymmetry parameter where polarisation states (**a,b**) x-linear, (**c,d**) y-linear, (**e,f**) left-circular, and (**g,h**) right-circular.

Figure 10 deals with the response of scattering intensity under x-LP, y-LP, R-CP, and L-CP to varying the scattering angle, the beam’s topological charge, and the asymmetry parameter. An increase in the aforementioned parameter leads to an expansion of the interaction surface of the TI sphere, thereby improving the scattering intensity. Flat areas in the figures of scattering intensity look like weak scattering responses, whereas sharp edges and peaks show strong scattering influences that come from the interaction between the NDLB and the TI spherical scatterer.

The scattered intensity is plotted by employing a stacked representation to illustrate separate scattering curves over discrete topological charges, i.e., v = 0,1,2,3,4,5, while the scattering angle θ is retained as a continuous variable θ∈[0,π]. These representations illustrate mode-dependent variation. Separate plots for linear polarisation (X, Y) and circular polarisation (LCP, RCP) represent polarisation-dependent response. This approach ensures consistency with the intrinsic discreteness of the topological charge v.

For Fig. 10, the asymmetry parameter modifies the characteristics of the incoming beam field, altering the efficiency of excitation for higher-order beam modes and stimulating the angular scattering pattern. When c = 0, the NDLB exhibits symmetrical characteristics, and the scattering response is inherently smooth. The amplification of the c parameter, i.e., c ≠ 0, induces asymmetric properties. This assistance produces interference phenomena regarding scattered field trends. The beam’s topological charge (v) excites internal field modes, enforcing mode compatibility with the resonant modes of the TI scatterer. As the size parameter increases, additional topology-based modes tune, thereby modifying the scattering efficiency. Various polarisation states also alter the excitation symmetry in TI scatterers, allowing polarisation to strengthen or diminish specific resonances.

In conclusion, the interaction of shaped-beam profile, topological charge, beam asymmetry factor, and polarisation-sensitive coupling yields the substantial differences toward size-based and polarisation-resolved scattering efficiency factor.

**Fig. 11:**
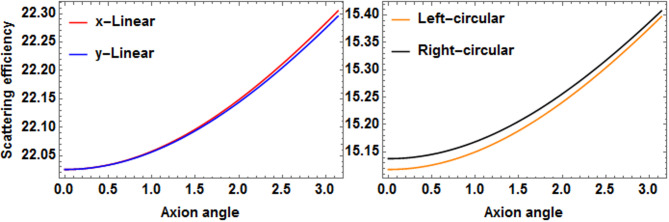
Total scattering efficiency of a TI sphere under Lommel beam illumination for axion angle ($\:{\theta\:}_{0}$) under various polarisation states, i.e., linear (x & y) and circular (left & right). The figure shows a nonlinear upswing because of axion-based magnetoelectric coupling and minimal chirality-induced asymmetry.

Figure 11 shows the scaling of total scattering efficiency with the axion angle $\:{\theta\:}_{0}$ of the TI spherical scatterer. The presented numerical results of Fig. 9 are physically consistent with axion-based scattering by a TI sphere illuminated by a NDLB. The total scattering efficiency as given in Eq. (6) increases nonlinearly and monotonically versus the axion angle $\:{\theta\:}_{0}$, reflecting boosted magnetoelectric coupling initiated by the axion factor. Since total scattering efficiency depends on the square of field amplitudes, a quadratic-like dependence on the axion angle is seen instead of stringent linear scaling. For linear polarisations, the approximately overlapping scattering curves validate the isotropy of the TI sphere, as orthogonal linear states amplify identical multipole responses. Contrary to that, the trivial separation between circular polarisation states, i.e., left and right, generates from axion-mediated magnetoelectric coupling, which proposes handedness-dependent interactions. These interactions are generated because of the spin angular momentum of CP fields.

**Fig. 12:**
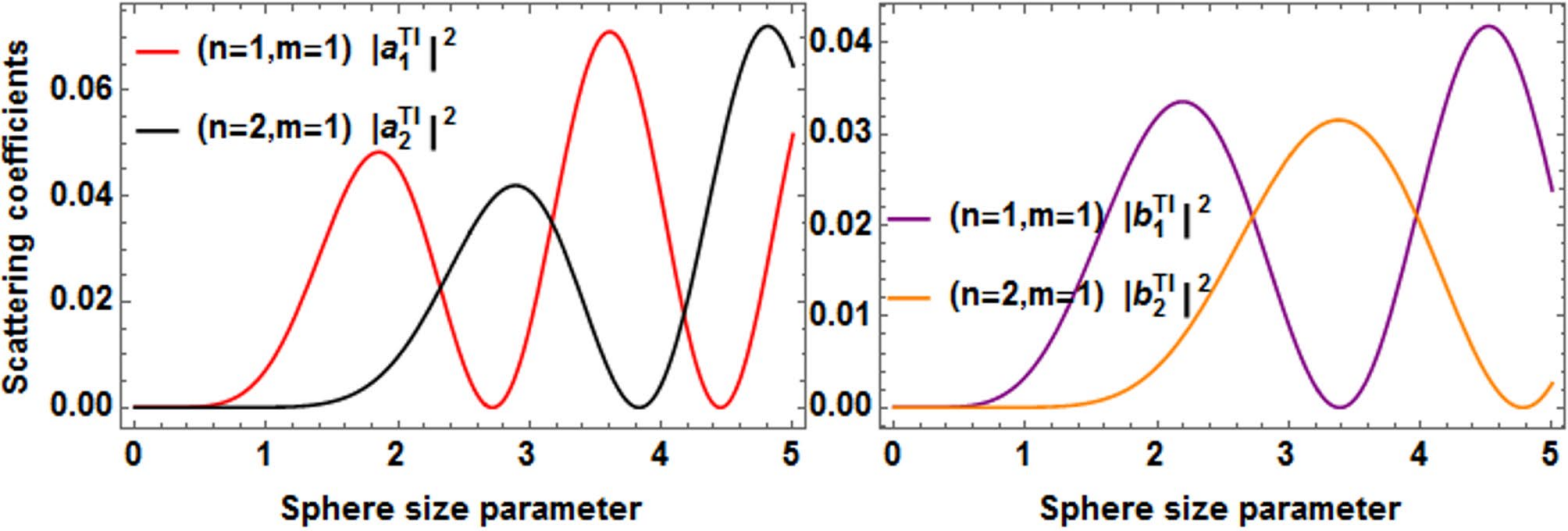
Scattering field coefficients for orders *n* = 1 and 2 as a function of dimensionless size parameter. The numerical results are simulated for a TI sphere illuminated by a linearly polarised diffraction-free Lommel beam with m = 1. The electric dipole coefficient ($\:{a}_{1}^{TI}$, solid red) consistently recedes the electric quadrupole ($\:{a}_{2}^{TI}$, solid black) across the size range $\:\approx\:$2.0–5.0. For the magnetic dipole coefficients, the quadrupole ($\:{b}_{2}^{TI}$, solid orange) is consistently exceeded by dipole ($\:{b}_{1}^{TI}$, solid purple) and becomes increasingly substantial with increasing size parameter.

Figure 12 shows the cross-polarised scattering field coefficients $\:{a}_{n}^{TI}$ and $\:{b}_{n}^{TI}$ under linearly polarised Lommel beam illumination for *n* = 1 and 2 as a function of the size parameter of the TI sphere. There is an oscillating excitation of electric and magnetic field modes. The TME effect plays a central role in cross-polarised scattering coefficients. It is obvious that there exist distinct dominant multipoles. For electric multipoles, i.e., $\:{a}_{n}^{TI}$, specifically the dipole mode at *n* = 1, it continually lacks the quadrupole at *n* = 2 across all the dimensionless size parameters. The figure also shows that the quadrupole entirely dominates over the electric one at around size parameter of 2.75 and 3.8, where the electric dipole nearly vanishes. However, for magnetic multipoles, i.e., $\:{b}_{n}^{TI}$, a size-based transition occurs. Here, quadrupole dominates for dimensionless sphere size parameter around 3.25, while dipole shows receding response.

Furthermore, for quantitative explanation, Table 1 shows the contributions of dominant multipoles to the cross-polarised response for size parameters, on the basis of the scattering coefficients.

**Table 1:** Dominance of various multipoles in cross-polarised scattering over dimensionless size parameter.

Sphere size parameter	Majorly contribution of multipoles	$\:{\left|{a}_{mn}^{TI}\right|}^{2}$ (m, *n*)=(1,1)	$\:{\left|{b}_{mn}^{TI}\right|}^{2}$ (m, *n*)=(1,1)	$\:{\left|{a}_{mn}^{TI}\right|}^{2}$ (m, *n*)=(1,2)	$\:{\left|{a}_{mn}^{TI}\right|}^{2}$ (m, *n*)=(1,2)
1.0	Electric dipole	0.200	0.050	0	0
1.5	Electric dipole	0.250	0.060	0.040	0.045
2.0	Electric dipole	0.240	0.040	0.020	0.025
3.5	Electric dipole+ quadrupole	0.110	0.020	0.040	0.045
4.0	Electric dipole+ quadrupole	0.090	0.030	0.060	0.065

For size parameter ≤ 2.0, dipoles lead the cross-polarised characteristics. However, for size parameter$\:\approx\:$3.5-4.0, quadrupoles become equivalent, incorporates further scattering channels.

The finest result is the axion-based cross-polarised scattering response, i.e., the involvement of cross-polarised scattering driven by the TME phenomena. This is generated from the axion-modified BCs. Unlike conventional dielectric spheres where cross-polarised scattering field coefficients are identically zero, the TI medium yields non-zero components controlled by θ, independent of the beam’s topological charge or asymmetry parameter. This decoupling offers a unique TME response absent in classical Mie theory.

Though alternative structured beam fields and nanophotonic avenues can achieve similar control, the diffraction-free Lommel beams offer a remarkably efficient pathway for far-field response and mode-selective stimulation of the TME signal. Indeed, other structured fields like polarised vector beams, vortex beams, Airy beams, and many more can also tune the polarisation characteristics, phase profile, and OAM distribution of light beams and, consequently, may influence electromagnetic interaction with topological materials. Particularly, vector beams can boost polarisation-sensitive coupling, while vortex beams introduce controllable OAM that can tune selection rules in relation to the scattering processes. However, the primary attributes of Lommel beams in the proposed work are their intrinsic asymmetric angular spectrum combined with asymmetry parameter c, which enables a noticeable mode-selective stimulation of multipolar resonances in the TI medium. This precise spectral asymmetry feature is not generally intrinsic to conventional vortex and/or diffraction-free Airy beams, where the angular spectrum is specifically more symmetric or controlled by various phase structures. Non-beam-based approaches, including metasurface engineering or near-field excitation, can tune the topological magneto-electric response by regulating field confinement and polarisation features. However, these techniques depend on primarily distinct excitation phenomena instead of free-space shaped beams.

## Discussion

The central finding of this work is not simply that a Lommel beam scatters differently from a Bessel beam at a topological insulator sphere, it is that the Lommel asymmetry parameter *c* functions as a tunable spectroscopic dial for the axion coupling itself. To appreciate why this matters, consider the challenge facing any experimental attempt to detect the TME effect optically: the cross-polarised scattering signal driven by the axion angle θ is superimposed on a much larger co-polarised Mie background^27^, and the two cannot be separated by choosing a different wavelength or a different sphere size. The co-polarised background is not a measurement artefact but an intrinsic consequence of the dominance of conventional Mie multipoles, which respond to any incident field irrespective of axion coupling. What can disentangle the TME signal from this background is selective multipole excitation, driving the modes that carry the axion signature while leaving the background largely unaffected. This principle is well established in the structured-light scattering literature: engineering the angular spectrum and polarisation state of the incident beam allows preferential coupling into chosen multipole orders, enabling isolation of weak resonant responses that would otherwise be overwhelmed^24,25^. The present work shows that *c* provides exactly this lever for the TME case. As *c* increases from zero, the azimuthal symmetry of the Lommel beam’s angular spectrum is progressively broken, redistributing beam-shape coefficient energy among multipole orders in a predictable, continuous way^27–29^. Because the cross-polarised coefficients $\:{a}_{n}$ and $\:{b}_{n}$ are mode-dependent, different multipole orders coupling to the axion term with different strengths, this redistribution changes the ratio of TME-driven to background scattering, and for appropriate values of *c* it amplifies the axion signature without requiring any change in material, geometry, or illumination wavelength. No cylindrically symmetric beam, Bessel, Laguerre-Gaussian, or otherwise, can perform this function: once topological charge and cone angle are fixed, the angular spectrum is fully determined and no further continuous degree of freedom remains to shift BSCs selectively among multipole orders^22,23,29^. The Lommel beam is therefore not a minor generalisation of the Bessel beam: it is a qualitatively different class of probe, one that converts a continuous structural parameter into a continuous spectroscopic handle on axion electrodynamics, making an otherwise experimentally buried topological signature legible^30^.

**Fig. 13:**
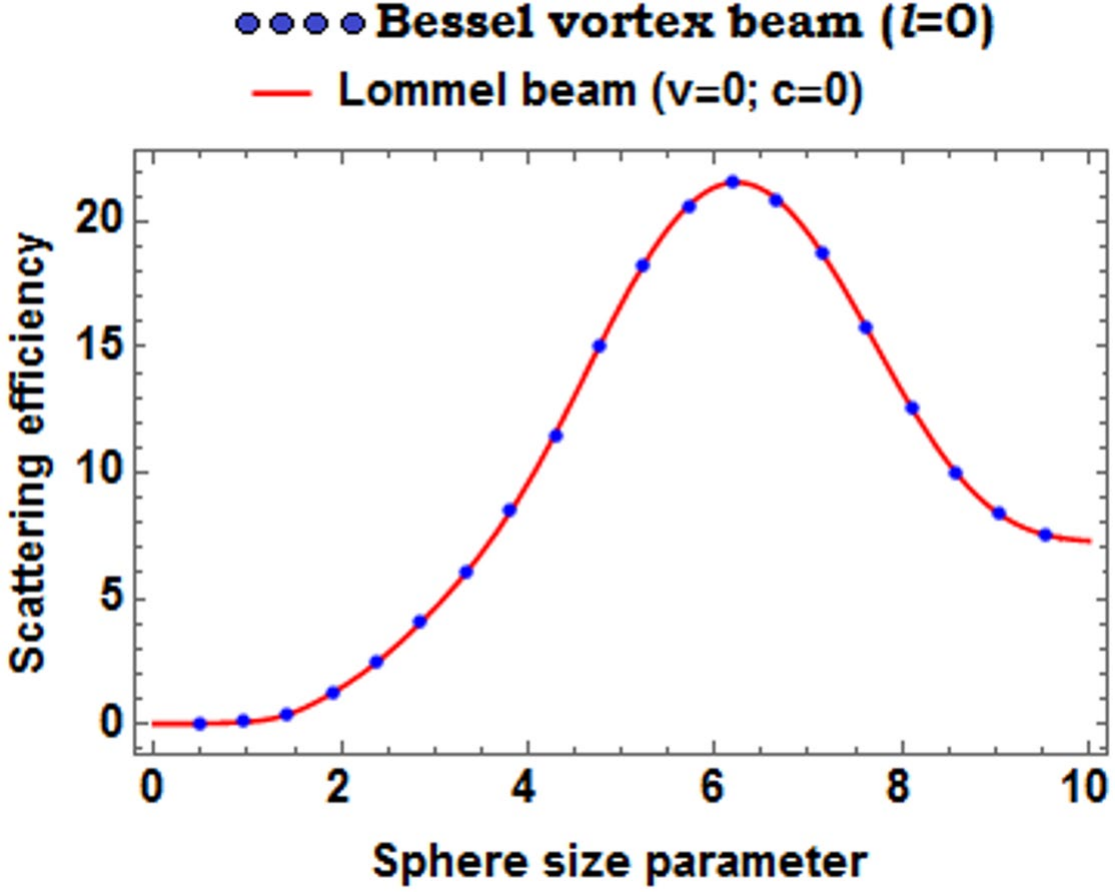
Comparison of the scattering efficiency for proposed work under the limiting case for NDLB *\:(v=0=c)* and its transformation to a Bessel vortex beam for *\:(l=0)* for the TI sphere.

To benchmark the numerical results, by adopting the special case, i.e., characterizing parameters of the Lommel beam *\:c=v=0*, the scattering efficiency $\:\left({Q}_{sca}\right)$ of the Lommel beam for the topological insulator sphere is plotted. The dimensionless size parameter is varied for *\:0-10*. For the same scenario, the topological insulator sphere is illuminated by a zeroth-order vortex Bessel beam. As the linear combination of the Bessel beam modes constitutes NDLB. It can be seen that excellent agreement exists under the limiting case of the Lommel beam and the vortex Bessel beam. This validation is displayed in Fig. 13.

Furthermore, by adopting the special case, i.e., the axion angle of the topological insulator $\:{\theta\:}_{0}=0$, which results in $\:\stackrel{\sim}{\alpha\:}=0$, and this yields auxiliary parameters $\:{\beta\:}_{1}={\beta\:}_{2}=1$, all the scattering field coefficients of the TI become null, $\:{a}_{n}^{TI}={b}_{n}^{TI}={c}_{n}^{TI}={d}_{n}^{TI}=0$. This special condition makes the scattering and transmitted coefficients regarding TI equal to traditional coefficients as found in^39^, excluding the product of BSCs of NDLB. Bessel beams verify the method. The asymmetry parameter offers uninterrupted control over multipole excitation, resulting in selectively exciting the axion-based cross-polarised scattering response while reducing the background scattering—a distinguished degree of freedom that Bessel beams lack.

A natural question arising from the Bessel beam validation (Fig. 13) is what Lommel beams specifically enable that a Bessel beam cannot. The answer lies in the asymmetry parameter c, which constitutes an additional continuous degree of freedom absent in standard Bessel or Laguerre-Gaussian beams. As c is varied from zero, the azimuthal symmetry of the incident field is progressively broken, inducing a redistribution of energy among multipole orders through the beam shape coefficients. This selective multipole excitation is not achievable with a Bessel beam, where the angular spectrum remains fixed by the topological charge and half-cone angle alone. For TI scatterers specifically, this matters because the cross-polarised coefficients $\:{a}_{n}^{cross}$ and $\:{b}_{n}^{cross}$are mode-dependent, different multipole orders couple differently to the axion term through the boundary conditions. The lowest-order multipoles that couple effectively to the axion-mediated electromagnetic term control the response of the cross-polarised component. For the dipolar modes, the principal contribution is established since they represent the substantial field overlap with the axion term, yielding the dominant cross-polarised signal. Higher-order modes, like the quadrupolar contribution, are weaker since they don’t stimulate because of the reduced excitation efficiency. However, they can become significant at higher frequencies. This behaviour is made clearer by explicitly mentioning that the observed cross-polarised response is led by dipoles under the specific parameters, with higher-order multipoles only adding insignificant corrections. By tuning c, one can preferentially excite multipoles that are most sensitive to the TME effect, effectively amplifying the cross-polarised signal for a given axion angle. The Lommel beam thus functions as a tunable probe of the topological surface states, offering a richer diagnostic capability than a fixed Bessel beam illumination.

To quantify the TME phenomena, we investigate the scattering asymmetry c by simulating the ratio of cross-polarised to co-polarised field coefficients. For a TI sphere furnished with a time-reversal symmetry-breaking surface gap illuminated by a diffraction-free Lommel beam, the intensity of the backscattered cross-polarised term can surpass the co-polarised scattered intensity by a factor proportional to $\:{\alpha\:}^{2}$, the square of the fine-structure constant. This ratio, stemmed from the interference of helicity in the Mie scattering coefficients, offers a distinct experimental signature apparent from ordinary dielectrics, where such cross-polarised improvement is absent.

Unlike a non-diffracting Bessel beam, a Lommel beam holds a tunable asymmetry term that breaks cylindrical symmetry. This permits it to specifically boost the cross-polarised scattering field coefficients induced by the TME characteristics that remain invisible toward the symmetric scattering response of a Bessel beam. A diffraction-free Lommel beam is well renowned for its asymmetry parameter c, which furnishes a tunable channel regarding multipole excitation. Different from the fixed cylindrical symmetry of a non-diffracting Bessel beam, this term permits the particular intensity or repression of specific electromagnetic multipole moments within the TI medium, making the sensitive cross-polarised spectrum of the TME phenomena experimentally comprehensible. Such tunability is not available with non-diffracting Bessel beams.

NDLB comprised asymmetric field distribution and higher-order angular spectrum. A Lommel beam can excite multipoles in a different way than a plane wave due to the following: it can stimulate various higher-order field modes. It also can boost incident field coefficients. By illuminating a TI with a NDLB generates a spatially structured term comprising **E**·**B**. The TME, i.e., $\:\left({\theta\:}_{0}\right)$ of TI links to this product, consequently producing a supplementary TME-based contribution towards induced dipole moments. Furthermore, the achieved results of (**E**·**B** ≠ 0) generates central zone of robust **E**·**B** toward beam’s cross-section due to unique spatial structure and non-diffracting features of Lommel beam.

To assess the experimental accessibility of the predicted effects, we note that the canonical three-dimensional topological insulator materials, Bi₂Se₃ and Bi₂Te₃, are characterised by a quantised axion angle$\:{\theta\:}_{0}$ = π (mod 2π) when time-reversal symmetry is preserved, corresponding to a topological magnetoelectric polarisability α = e²/2hc in Gaussian units. Upon application of a modest external magnetic field ( ≲ 0.1 T) or through magnetic doping, time-reversal symmetry is broken and $\:{\theta\:}_{0}$ departs from this quantised value, allowing continuous tuning of the TME response. The sphere size parameters explored here (ka ≈ 2–5) correspond to physical radii in the range of 150–400 nm for visible or near-infrared illumination (λ ≈ 600–800 nm), which is well within the fabrication capability of existing colloidal and lithographic synthesis methods for Bi₂Se₃ nanoparticles. The predicted nonlinear increase in total scattering efficiency with $\:{\theta\:}_{0}$ (Fig. 11) and the onset of measurable cross-polarised intensity at moderate axion angles therefore constitute realistic observational targets for angle-resolved polarimetric scattering experiments. Structured Lommel beam illumination, now routinely generated using spatial light modulators, provides the beam control required to exploit the multipole selectivity demonstrated here.

Our analytical model and achieved numerical results for the Lommel beam asymmetry parameter and topological charge are consistent under a special condition with the Bessel beam findings. So, it indicates that Lommel beams^26^ are quite similar to Bessel beams^41^. The variation in the configuration parameters of the Lommel beam also excites the interference effect between the incident, scattered, and transmitted electromagnetic fields. Furthermore, it’s important to note that TME contributes to the scattering response toward the TI medium. The following are the most important physical findings: Topological-based asymmetric scattering characteristics, mode dependence, and soundness of the TI axion coupling. A Lommel beam illumination yields a different intensity distribution in the far field than the typical scattering response of a dielectric sphere due to cross-polarised field components. We discover that particular scattering resonances can be tuned by varying the beam’s asymmetry, a capability not achievable with conventional Gaussian or Bessel beam illumination. The Lommel beam basis makes the scattering signatures of the axion coupling term stronger. This proves that structured light is very fine to evaluate the controllable scattering fingerprints on the TI scatterer. Numerical results display that characterizing beam parameters rigorously impacts the scattering pattern of electric fields. These results offer new potentials for practical photonics applications and serve as an explanation for how the interaction of structured beams works in a complicated optical atmosphere. Future studies could explore the impact of Lommel beams in nonlinear optics, particle sizing, metamaterial structures, laser optics, optical trapping, and guiding.

## Data Availability

All data that support the findings of the study has been included into the manuscript.
